# Lemon Essential Oil-Enriched Alginate–Gelatin Coating Preserves Bioactive Quality in Strawberries: Extraction Protocol Effects and an Exploratory Modeling Approach

**DOI:** 10.3390/foods15213834

**Published:** 2026-10-27

**Authors:** Tugca Bilenler Koc, Ulkuhan Bagıs, Huseyin Karakaya, Ihsan Karabulut

**Affiliations:** 1 Department of Food Engineering, Faculty of Engineering, Inonu University, Malatya 44280, Turkey; huseyin.karakaya@inonu.edu.tr (H.K.); ihsan.karabulut@inonu.edu.tr (I.K.); 2 Food Processing, Food Quality Control and Analysis Program, Zara Ahmet Çuhadaroğlu Vocational School, Cumhuriyet University, Sivas 58700, Turkey; ulkuhanbagis@cumhuriyet.edu.tr

**Keywords:** strawberries, edible coating, postharvest quality, phenolic compounds, support vector regression

## Abstract

Strawberries (*Fragaria × ananassa*) deteriorate quickly during ambient storage. This study evaluated sodium alginate–gelatin coatings, with and without lemon essential oil (LEO), on strawberry quality over five days of storage, and how the extraction protocol used to assess quality affects the resulting values. Fruits were divided into uncoated control (K), edible film (F), and LEO-enriched film (FE) groups and extracted using three solvents and two homogenization methods. Weight loss, color, microbial counts, 2,2-diphenyl-1-picrylhydrazyl (DPPH) radical scavenging activity, total phenolic content (TPC), and individual phenolics were assessed. The FE coating was the most effective: weight loss reached 18.94% versus 28.99% in the control, and yeast and mold counts reached 1.10 versus 5.33 log CFU/g; *Escherichia coli* was not detected, and FE retained color closer to initial values. DPPH and TPC losses were 8.2% and 3.7% under FE versus 33.5% and 14.7% in the control, but LEO itself gave DPPH and TPC signals while the coating matrix did not, so part of this antioxidant activity reflects the oil rather than protected fruit phenolics. Individual phenolics were nearly eliminated in uncoated fruit, whereas coated fruit lost 30% or less, with similar retention in F and FE. Extraction protocol strongly affected the measured values: Ultra-Turrax outperformed sonication, and water gave the highest DPPH and TPC values and water–ethanol the lowest. An exploratory support vector regression model, not validated on independent samples, reached R^2^ ≥ 0.85 for 8 of 14 phenolics.

## 1. Introduction

Strawberries (*Fragaria × ananassa*) are among the most highly demanded fruits worldwide because of their flavor, attractive color, and nutritional value. They are a good source of vitamin C, anthocyanins, minerals, and phenolic compounds [1,2]. Despite their nutritional value, strawberries deteriorate quickly after harvest because of their delicate tissue, high respiration rate, and rapid moisture loss [1]. These weaknesses make them prone to mechanical damage and microbial infection, particularly fungal decay, so their postharvest shelf life is short [1,2]. Under them extremely susceptible to mechanical damage and microbial infections, particularly fungal decay, and their postharvest shelf life is therefore short [1,2]. Cold storage, modified atmosphere packaging, and synthetic fungicides are used to slow spoilage and reduce postharvest losses, which can reach 50% [3]. Growing consumer demand for eco-friendly, residue-free, and safe food has increased interest in sustainable preservation technologies, and biopolymer-based edible coatings are among the most studied options [1,3,4].

By forming a thin semi-permeable barrier on the fruit surface, edible coatings limit the exchange of gases such as oxygen and carbon dioxide, reduce respiration rate, and reduce moisture loss through transpiration, thereby maintaining product quality [3,5]. Sodium alginate (SA), one of the most widely used biopolymers in the food industry, has excellent film-forming capacity, is non-toxic, and is biocompatible [2,6,7]. Although SA can preserve the appearance and extend the shelf life of foods, it is highly hydrophilic and does not form a sufficient water vapor barrier on its own [5,6,8]. Blending SA with other proteins or polymers is a common way to improve its water resistance [6].

Gelatin (G) is a water-soluble protein derived from collagen. It is flexible and has good film-forming, stabilizing, and thickening properties [5]. Adding gelatin to the SA matrix gives a composite SA:G matrix with greater elasticity, better structural integrity, and gas (O_2_ and CO_2_) barrier properties [5,6]. The high hydrophilicity of polysaccharide–protein composite films, however, remains an obstacle to optimizing water barrier performance on fruit surfaces [9,10,11,12].

Incorporating hydrophobic plant essential oils into the polymer matrix can reduce this limitation and give the coating antimicrobial and antioxidant activity [5]. Lemon essential oil (LEO) is rich in terpenes and terpenoids, has antimicrobial and antioxidant activity, and is generally recognized as safe (GRAS) [5,13]. Adding LEO to the SA:G matrix introduces hydrophobic domains into the hydrophilic network. These domains lengthen the diffusion path of water molecules and have been associated with lower water vapor permeability [14]. LEO components are reported to inhibit yeast and mold growth and to slow the oxidative breakdown of phenolic compounds and ascorbic acid in the fruit [15,16].

Edible coatings have been tested on strawberries in numerous studies, many of which apply a single formulation, monitor a single storage period, and quantify bioactive compounds with a single extraction protocol [1,3,14]. Such designs describe the outcome of one coating under one analytical procedure. They cannot show how much of the reported change in phenolic content depends on the coating and how much depends on the way the phenolics were extracted. This gap matters in two ways: producers need quantitative guidance beyond the single combinations tested in individual studies, and researchers face the same limitation when generalizing findings to untested conditions. Machine learning approaches, particularly support vector regression (SVR), are well suited to model nonlinear relationships between processing variables and phenolic stability directly from experimental data [17], but their application to postharvest coating research remains limited. Recent reviews note that machine learning-based shelf-life prediction for fruits and vegetables is still an emerging area with few applied studies [18]. Fewer studies have combined such modeling with a systematic comparison of extraction solvents and homogenization techniques, although solvent choice alone can substantially change phenolic recovery and antioxidant capacity in berry species, including strawberry [19]. This study therefore combines three elements in one experimental design: (i) an SA:G coating enriched with LEO, compared with an SA:G coating without LEO and an uncoated control; (ii) a systematic comparison of three extraction solvents and two homogenization methods for phenolic quantification; and (iii) an exploratory SVR analysis of how coating treatment, storage day, extraction solvent, and homogenization method relate to individual phenolic concentrations. This design allows the effect of the coating to be examined alongside the effect of the extraction protocol, which single-formulation and single-protocol studies cannot do.

The study addressed two questions with equal weight: how the coating affects the retention of bioactive compounds in strawberries during ambient storage, assessed through weight loss, color, microbial quality, antioxidant activity, and individual phenolic content; and which combination of extraction solvent and homogenization method recovers strawberry phenolics most effectively. SVR and principal component analysis (PCA) were used as exploratory tools to describe and rank the contributions of coating treatment, extraction solvent, homogenization method, and storage day; the SVR models were not tested on independent samples.

## 2. Materials and Methods

### 2.1. Materials and Chemicals

Strawberry fruits (*Fragaria* × *ananassa* Duch.) were purchased from a local market in Malatya, Türkiye. Fruits were visually inspected, and samples exhibiting uniform size, maturity, and freedom from mechanical damage were selected for the study. Lemons (*Citrus limon*) were purchased from a local market in Malatya, Türkiye, and the peels were used for oil extraction.

Ethanol was purchased from Merck (Darmstadt, Germany). Sodium alginate, gelatin, 2,2-diphenyl-1-picrylhydrazyl (DPPH), Folin–Ciocalteu reagent, Sabouraud Dextrose Agar (SDA), gallic acid, buffered peptone water (BPW), and all chemical standards were purchased from Sigma-Aldrich (Steinheim, Germany) unless otherwise specified.

### 2.2. Specifications of Lemon Essential Oil (LEO)

Lemon essential oil (LEO) was obtained in our laboratory by hydrodistillation for 3 h using a Clevenger-type apparatus and stored at 4 °C until use. Specific gravity was determined at 25 °C and refractive index at 20 °C [20]. The chemical composition of LEO was determined with a Shimadzu GC-MS QP2010 Plus system (Shimadzu, Kyoto, Japan) fitted with a DB-WAX capillary column (60 m × 0.25 mm i.d., 0.25 µm film; J&W Scientific, Folsom, CA, USA), following Karakaya et al. (2022) [21]. Helium was used as the carrier gas at 1 mL/min. The oven was held at 40 °C for 5 min, raised to 240 °C at 3 °C/min, and held for 15 min. The injector, transfer line, and ion source were set to 250 °C. Mass spectra were recorded in electron ionization mode at 70 eV over *m*/*z* 45–450. One microliter of oil diluted in hexane (1:10 *v*/*v*) was injected with a split ratio of 1:100. Compounds were identified by comparing their mass spectra with the Wiley (John Wiley & Sons, Hoboken, NJ, USA), NIST (National Institute of Standards and Technology, Gaithersburg, MD, USA), and FFNSC (Shimadzu, Kyoto, Japan) libraries and their retention indices, calculated from a C8–C40 n-alkane series, with NIST WebBook values. Relative percentages were calculated from peak areas, and analyses were run in duplicate.

### 2.3. Preparation of Coating Formulations

The coating formulation was prepared by dissolving SA (1.5 g) in 70 mL of distilled water under continuous stirring at 70 °C for 2 h. In a separate vessel, gelatin (1.5 g) was dissolved in 30 mL of distilled water at 40 °C for 1 h. The gelatin solution was subsequently combined with the sodium alginate solution, and the mixture was stirred for 25 min followed by incubation in a water bath for 40 min. Glycerol (0.5% *w*/*v*) was added as a plasticizer, the mixture was stirred at 25 °C for 10 min. Lemon essential oil (3% *v*/*v*) was then incorporated into the formulation, while Tween 80 (0.5% *v*/*v*) was added as an emulsifying agent. The LEO concentration was selected from the literature and confirmed in preliminary trials with 1%, 3%, and 5% (*v*/*v*): at 1%, microbial spoilage progressed rapidly, and 5% adversely affected the sensory quality of the fruit; 3% was therefore used. Following the addition of lemon essential oil and Tween 80, the mixture was stirred at room temperature for 30 min and subsequently maintained under continuous agitation at 25 °C for 120 min to ensure complete homogenization before application. A blank formulation was also prepared by following the same procedure without the LEO addition step; this formulation served both as the coating applied to the F treatment group and as a coating-matrix control in the extraction [22,23].

### 2.4. Coating Application and Storage Conditions

Selected fruits were immersed in distilled water containing 0.1% sodium hypochlorite for 2 min for surface disinfection, rinsed three times with distilled water, and air-dried at room temperature. The fruits were divided into three experimental groups: uncoated control fruits (K), fruits coated with the SA:G formulation without LEO (F), and fruits coated with the SA:G formulation containing LEO (FE). Strawberries were immersed in the respective coating solutions for 45 s, allowed to drain, and subsequently air-dried at room temperature for 2 h [22]. Both coated and uncoated fruits were stored at room temperature (22 °C) for 5 days; relative humidity was not monitored. For the analysis, samples were taken randomly from the relevant sample group on days 0, 3, and 5 of storage.

### 2.5. Weight Loss Determination

For each treatment group, measurements were performed in triplicate, with six fruits per replicate (n = 18). The weight loss of the strawberries was determined gravimetrically according to the following formula [14]:

$$Weight\;loss\;\left(\%\right)=\frac{M_{i}-\;M_{t}}{M_{i}}\times 100$$

where *M_i_* represents the initial weight of the strawberries and *M_t_* represents the weight recorded at the corresponding storage time.

### 2.6. Fruit Color Measurement

Strawberry surface color was assessed with a portable colorimeter (CR-10, Konica Minolta, Tokyo, Japan), recording the L*, a*, and b* coordinates [24]. For each fruit, three measurements were taken at equally spaced points around the equatorial region, and their average was used in the subsequent statistical analysis.

### 2.7. Microbiological Analysis

Total yeast and mold counts were determined as previously described [1] with slight modifications [1]. Briefly, 10 g of strawberry sample was homogenized with 90 mL of sterile saline solution (0.9% *w*/*v*). The resulting homogenate was serially diluted to 10^−6^ using sterile saline solution. Aliquots (0.5 mL) from the last three dilutions were plated onto SDA, and plates were incubated at 25 ± 1 °C for 5 days. To ensure that yeast colonies were not missed, their growth on plates was checked and recorded daily. The colonies were counted as yeast and mold separately, and the results were expressed as log CFU/g after correction for the corresponding dilution factor [5].

For *Escherichia coli* enumeration, samples were homogenized in BPW at a 1:9 (*w*/*v*) ratio, followed by preparation of decimal serial dilutions. *E. coli* was quantified on Eosin Methylene Blue (EMB) agar after 24 h of incubation at 37 °C, with counts expressed as log CFU/g [25]. One milliliter of the 10^−1^ dilution was plated, giving a detection limit of 1.0 log CFU/g; samples with no colonies were reported as not detected (<1.0 log CFU/g).

### 2.8. Extraction of Strawberry Samples

Strawberry samples subjected to different coating treatments (K, F, and FE) were extracted using three solvent systems as follows: water (100%), ethanol (100%), and water–ethanol (50:50 *v*/*v*). In the extraction process, two homogenization methods, mechanical (Ultra-Turrax, U) and ultrasonic (water bath, S), were used. Five grams of sample was mixed with 10 mL of the extraction solvent. For mechanical extraction, the mixture was homogenized at 12,000 rpm for 5 min in an ice bath using an Ultra-Turrax (IKA, Staufen, Germany). For ultrasonic extraction, the mixture was placed in an ultrasonic water bath (Sonorex Super RK 514 H, Bandelin Electronic, Berlin, Germany; 13.5 L, ultrasonic peak power 2 × 450 W) operating at 35 kHz for 10 min. The bath temperature was maintained at 20 °C with ice packs throughout the treatment, and the bath heater was not used. After extraction, the mixture was centrifuged at 5580× *g* for 15 min at 4 °C using a refrigerated centrifuge (SL 16R, Thermo Fisher Scientific, Osterode, Germany), and the entire supernatant was transferred to a 25 mL volumetric flask. The pellet was re-extracted with 5 mL of the same solvent, vortexed for 2 min, and centrifuged under identical conditions, and the entire supernatant was again collected in the same flask. This re-extraction was performed three times (giving four extraction cycles in total). The combined supernatants were made up to 25 mL with the corresponding solvent. The extracts were then filtered through a 0.45 µm nylon membrane filter (Lubitech, Shanghai, China), transferred into amber vials, and stored at 4 °C until analysis. The obtained extracts were used for the determination of antioxidant activity (DPPH), total phenolic content (TPC), and individual phenolic compounds. Alongside the strawberry samples, 1 mL each of the blank coating solution and LEO (diluted to 30 µg/mL) were subjected to the same extraction procedure across all solvent and homogenization combinations, allowing the intrinsic contribution of the coating matrix and the essential oil to be assessed independently of the fruit.

### 2.9. Antioxidant Activity

The radical scavenging activity of strawberry extracts was determined using the DPPH assay according to the method of Brand-Williams et al. (2005) with minor modifications [26]. Briefly, a DPPH stock solution was prepared in 80% methanol. Then, 100 µL of strawberry extract was mixed with 1.9 mL of the DPPH solution and incubated at room temperature for 30 min in the dark. Following incubation, the absorbance of the reaction mixture was measured at 517 nm using a UV–Vis spectrophotometer (Shimadzu, Kyoto, Japan). The DPPH radical scavenging activity was calculated according to the following formula [26]:

$$Inhibition\;\left(\%\right)=\frac{A_{blank}-A_{sample}}{A_{blank}}\times 100$$

where *A_blank_* is the absorbance of the control solution containing all reagents except the sample, and *A_sample_* is the absorbance of the reaction mixture containing the extract.

In addition to the K, F, and FE strawberry extracts, the corresponding extracts of the blank coating solution and LEO were analyzed under the same conditions to determine their individual contribution to the measured radical scavenging activity.

### 2.10. Total Phenolic Content

TPC of strawberry extracts was determined using the Folin–Ciocalteu method described by Kraujalytė et al. (2013), with slight modifications [27]. Briefly, 100 µL of extract was mixed with 1 mL of 0.2 N Folin–Ciocalteu reagent and 400 µL of distilled water. After 4 min, 1 mL of 7% sodium carbonate (Na_2_CO_3_) solution was added to the mixture. The reaction mixture was then incubated in the dark at room temperature for 90 min. Absorbance was measured at 725 nm using a UV–Vis spectrophotometer. The TPC was quantified using a calibration curve prepared with gallic acid standard solutions and expressed as mg gallic acid equivalents per 100 g fresh weight (mg GAE/100 g fw) [27]. As with the antioxidant activity assay, TPC was also determined for the blank coating solution and LEO extracts to distinguish their contribution from that of the fruit. This analysis was intended to show qualitatively whether LEO itself gives a Folin–Ciocalteu response, not to quantify its contribution to fruit TPC. LEO and fruit values are therefore reported on different bases (per gram of neat oil and per 100 g fresh weight, respectively) and should not be compared numerically.

### 2.11. HPLC Analysis of Phenolic Composition

HPLC analyses were performed using a Shimadzu HPLC system equipped with an autosampler (SIL-20A HT), column oven (CTO-10AS VP), degasser system (DGU-20A5R), gradient pump (LC-20AR), diode-array detector (DAD; SPD-M20A), refractive index detector (RID-10A), and LC solution software for instrument control and data acquisition. Individual phenolic compounds were determined according to the method of Campbell and Padilla-Zakour (2013) [28]. A 20 µL aliquot of each extract was injected into an ODS-3 column (250 × 4.6 mm, 5 µm; GL Sciences Inc., Tokyo, Japan). The column temperature was maintained at 25 °C, and the mobile phase flow rate was set at 1.0 mL/min. Chromatographic separation was achieved using a binary mobile-phase system consisting of solvent A (0.1% phosphoric acid in ultrapure water) and solvent B (0.1% phosphoric acid in HPLC-grade acetonitrile). The gradient elution program was initiated with 92% A and gradually decreased to 89% A at 4 min, 65% A at 25 min, 40% A at 30 min, held at 40% A for 10 min, and then returned to 65% A at 45 min, 89% at 50 min, and finally the initial conditions (92% A) at 55 min. Gallic acid, catechin, epicatechin, epigallocatechin, procyanidin B1, and procyanidin B2 were detected at 280 nm; caffeic acid, ferulic acid, *p*-coumaric acid, neochlorogenic acid, and chlorogenic acid at 320 nm; and quercetin-3-glucoside, kaempferol-3-glucoside, and rutin at 360 nm. Phenolic compounds were identified by comparison with authentic standards. Quantification was performed using calibration curves prepared from six concentrations of the corresponding standards. The concentration of each compound was calculated from the calibration curve of its own authentic standard and expressed as mg/100 g fresh weight (fw) [28]. The same chromatographic procedure was applied to the blank coating solution and LEO extracts to determine whether the individual phenolic compounds quantified in strawberry extracts were also present in the coating matrix or the essential oil itself. Under the same chromatographic conditions, the HPLC method was validated according to the ICH Q2(R1) [29] guideline in a previous study [30]. Concentrations below the LOQ were retained as calculated from the calibration curve.

### 2.12. Support Vector Regression (SVR) Modeling of Phenolic Stability

SVR with a radial basis function (RBF) kernel was used to model the relationship between experimental variables and individual phenolic compound concentrations. The categorical predictors (coating treatment, extraction solvent, and homogenization method) were converted to numeric form using one-hot encoding, while storage day was treated as a continuous numeric variable. All resulting features were then subjected to z-score standardization (mean = 0, SD = 1), since RBF kernel performance depends on the relative scale of the input variables. Both the encoding and the standardization were implemented as steps within a single scikit-learn pipeline together with the SVR estimator, rather than applied once to the full dataset beforehand. As a result, within each outer fold of the nested cross-validation described below, these transformations were fit only on that fold’s training data and then applied to its held-out test fold, preventing information from the test fold from influencing the scaling or the model. Hyperparameter selection and performance estimation were kept strictly separate to avoid the optimistic bias that arises when the same cross-validation folds are used for both purposes [31]. A nested cross-validation scheme was applied for this reason: the dataset was first divided into five outer folds, with four folds used for model building and the remaining fold held out entirely for testing. Within each of these four-fold training sets, a second, inner five-fold cross-validation was carried out to search for the combination of C, ε, and γ that gave the best fit, using the following search space (C ∈ {0.1, 1, 10, 100, 1000}; ε ∈ {0.01, 0.1, 0.5, 1.0}; γ ∈ {“scale”, “auto”, 0.01, 0.1, 1}). The model built with the best inner-fold parameters was then evaluated once on the outer test fold, which had played no part in either training or parameter selection. This process was repeated across all five outer folds, and the resulting five R^2^, RMSE, and MAE values were averaged to estimate how each model performs on unseen data.

The RBF kernel is defined as:

$$k\;\left(x_{i},x\right)=\exp\left(-\frac{∥x-x_{i}{∥}^{2}}{2\sigma^{2}}\right)$$

The SVR prediction function is:

$$f(x)=\sum_{i=1}^{n}(\alpha_{i}-\alpha_{i}^{*})\;k(x_{i},x)+b$$

where *α_i_* and α_i_* are the support vector coefficients, *k*(*x_i_*, *x*) is the kernel function, and b is the bias term.

Observed-versus-predicted comparisons are based on out-of-fold predictions obtained from the outer test folds of the nested cross-validation described above, so that no point reflects a prediction made on data the corresponding model had already seen. For interpretation purposes, such as the permutation-based feature importance analysis, a final model for each compound was fit on the complete dataset, with hyperparameters selected via the same grid search procedure described above, applied to the full set of 54 samples. This final model is used only to characterize how coating treatment, extraction solvent, homogenization method, and storage day relate to phenolic concentration; its performance is not reported separately because the nested cross-validation results already estimate it. Permutation-based feature importance was computed with 30 repeats per feature, using R^2^ as the scoring metric. Importance was computed by permuting each original categorical or numeric variable (coating treatment, extraction solvent, homogenization method, storage day) as a whole prior to one-hot encoding, rather than permuting individual one-hot encoded dummy columns separately; this avoids the distortion that permutation importance is known to introduce when applied to correlated or encoded dummy variables. Because this analysis was based on a single model fit to the complete dataset, variability in feature importance across resampling folds was not assessed; the reported percentages should be interpreted as a single-model summary rather than as estimates with associated uncertainty.

### 2.13. Statistical Analysis

Analyses were performed on a full factorial set of n = 54 experimental units, corresponding to three coating treatments × three extraction solvents × two homogenization methods × three storage days (3 × 3 × 2 × 3 = 54), with each combination represented by a single measured value rather than by independent biological or analytical replicates. Consequently, the mean ± SD values reported for pooled comparisons, such as across solvents or homogenization methods, reflect variability across these structural combinations rather than true replicate error. Weight loss and color were assessed separately with independent replicates (n = 18 and n = 4, respectively, as described above), so their standard deviations reflect genuine replicate variance.

Statistical evaluations were carried out by analysis of variance (ANOVA) using SPSS (version 16.0, SPSS Inc., Chicago, IL, USA), with mean differences assessed by Duncan’s multiple range test at a significance level of 0.05. Given the large number of dependent variables examined in this study, Duncan’s test does not correct for family-wise error, and results involving smaller mean differences should accordingly be interpreted with caution. This applies in particular to the DPPH, TPC, and individual phenolic comparisons, which span a large number of simultaneous pairwise comparisons across multiple experimental factors (coating treatment, extraction solvent, homogenization method, and storage day) and are therefore presented as exploratory rather than confirmatory.

Heatmaps and Principal Component Analysis (PCA) were performed using R software (version 4.6.0; R Foundation for Statistical Computing, Vienna, Austria). For the heatmap, concentrations were z-score normalized, and samples and compounds were clustered hierarchically using Euclidean distance and complete linkage (the default settings of the pheatmap package). PCA was performed on mean-centered and autoscaled concentrations of the 14 phenolics using the prcomp function. Group ellipses were drawn with the stat_ellipse function of the ggplot2 package (type = “norm”, level = 0.95) for exploratory visualization only and were not used for statistical inference. All violin plots were generated in Python 3 (Python Software Foundation, Wilmington, DE, USA) using the matplotlib library.

## 3. Results and Discussion

### 3.1. Specifications of Lemon Essential Oil (LEO)

The LEO had a specific gravity of 0.8537 ± 0.001 and a refractive index of 1.4738 ± 0.004. The specific gravity was very close to the value reported by Golmakani and Moayyedi (2015) [20] for lemon peel oil (0.854 ± 0.006 at 25 °C) and slightly higher than the range reported by Gölükcü et al. (2024) for lemon oils (0.8423–0.8471 g/mL) [32]. The refractive index was close to the range of 1.4740–1.4747 reported by Gölükcü et al. (2024) [32] and agreed with it within the measurement uncertainty. The GC-MS analysis identified seven compounds, which accounted for 96.91% of the oil: limonene (72.85%), γ-terpinene (10.14%), β-pinene (5.73%), β-myrcene (2.47%), α-pinene (1.96%), Z-citral (1.92%), and sabinene (1.84%). Limonene was the main component, as is typical for lemon oils, and this composition is consistent with that reported by Gök et al. (2015) [33] for lemon oils obtained by different extraction methods.

### 3.2. Weight Loss

**Figure 1:**
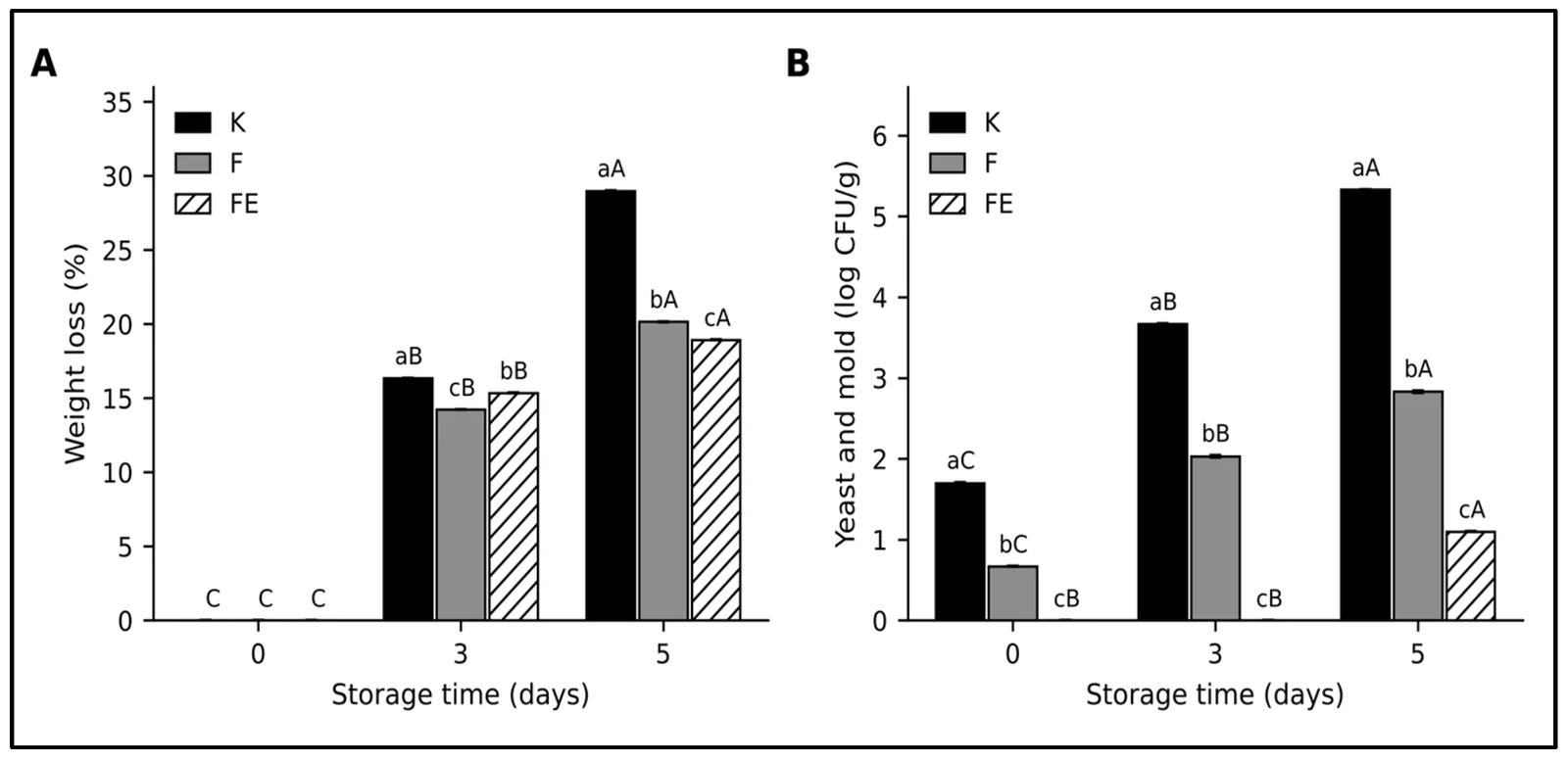
Changes in weight loss (%) (**A**) and yeast and mold counts (**B**) of strawberries during storage at 22 °C for 5 days. Bars show mean ± SD (n = 18 for **A**). Different lowercase letters indicate significant differences among treatments within the same storage day, and different uppercase letters indicate significant differences among storage days within the same treatment (*p* < 0.05). No bar indicates that no yeast or mold growth was detected. (K, uncoated fruits as control; F, coated fruits with SA:G; FE, coated fruits with SA:G:LEO).

The weight loss observed in fresh strawberries during storage is primarily associated with water vapor migration driven by physiological respiration and transpiration [18,34,35]. All sample groups showed an upward trend in weight loss throughout storage, but the applied coating formulations significantly (*p* < 0.05) slowed this loss. As shown in Figure 1A, the uncoated control (K) reached the highest weight loss by the end of storage, while fruit coated with the SA:G matrix (F) lost less (Table S1a,b). This protective effect is consistent with reports describing biopolymer coatings as a semi-permeable barrier on the fruit surface that can limit moisture diffusion and slow respiration [18,34]. Similar studies have linked this delay in weight loss to a reduction in water vapor permeability conferred by the coating [36], although permeability itself was not measured in the present study. On day 3, weight loss was lowest in F (14.23%), followed by FE (15.35%) and K (16.35%); on day 5, it was lowest in FE (18.94%), followed by F (20.16%) and K (28.99%). Incorporating LEO into a gelatin-based film has been shown to reduce water vapor permeability by over 20% while increasing surface hydrophobicity [37], which may explain the small additional protection observed in the FE-coated fruit on day 5; however, water vapor permeability, contact angle, and fruit respiration rate were not measured in this study, so this explanation was not tested.

### 3.3. Change in Fungal Counts

Because of their vulnerable epithelial tissue and high soluble sugar content, strawberries provide a favorable environment for yeast and mold growth during room-temperature storage. Yeast and mold counts differed significantly (*p* < 0.05) among treatments on every storage day, with the FE group showing consistently lower microbial loads than both F and K (Table S2a,b). The yeast and mold load in the control (K) group was 1.7 log CFU/g at the start of storage and increased sharply to 3.67 and 5.33 log CFU/g by day 3 and day 5, respectively (Figure 1B). In the group coated with the SA:G film (F), counts started lower (0.67 log CFU/g on day 0) and the increase was slower, reaching 2.83 log CFU/g by day 5. This partial inhibition may reflect the physical barrier formed by the coating, which has been reported to restrict oxygen availability at the fruit surface and create conditions less favorable for aerobic spoilage organisms [36]; oxygen permeability and respiration rate were not measured here, so this explanation was not tested. The LEO-enriched film (FE) showed the greatest resistance to microbial spoilage: yeast and mold growth remained undetectable through day 3, and counts reached only 1.1 log CFU/g by day 5. This additional effect may be related to the terpene components of essential oils, which are reported to penetrate microbial cell membranes and disrupt cellular integrity [34,35]; the antimicrobial activity of the LEO used here was not tested separately. *E. coli* was not detected in any sample, including the uncoated control, at any storage day (Table S3). Because no growth occurred even in the control, this analysis cannot show a coating effect on this organism. The absence of *E. coli* may reflect the surface disinfection applied to all fruit before treatment (Section 2.4) or the absence of initial contamination.

### 3.4. Fruit Color

Color parameters (L*, a*, b*) declined progressively in all three groups during storage, but the extent of this decline depended strongly on coating treatment (*p* < 0.001 for treatment, storage day, and their interaction, for all three parameters) (Table S4a–d). At day 0, no significant differences were found among K, F, and FE (*p* > 0.05), indicating that the initial fruit color was comparable across groups before storage began. By day 5, the uncoated control showed the sharpest loss of brightness and redness, with L* falling from 35.80 to 23.18 and a* from 25.82 to 16.73. Both coatings limited this decline, and the LEO-enriched film (FE) performed best, retaining L* at 28.20 and a* at 19.48; the plain SA:G film (F) fell between the two (L* = 27.08, a* = 18.28). A similar pattern held for b*, where FE- and F-coated fruit (14.72 and 14.50, respectively) remained closer to their initial values than the control (11.43).

This decline in lightness and redness during ambient storage is consistent with reports linking strawberry color loss to oxidative degradation of anthocyanins and the formation of brown pigments through polyphenol oxidase and peroxidase activity, both of which accelerate with storage time and temperature [35]. The slower decline observed in coated fruit, and particularly in the LEO-enriched coating, matches a pattern reported for other essential oil-enriched coatings, where the additional barrier and antioxidant effect of the oil helped delay water loss and enzymatic browning relative to a plain coating or an uncoated control [38].

### 3.5. Antioxidant Activity

**Figure 2:**
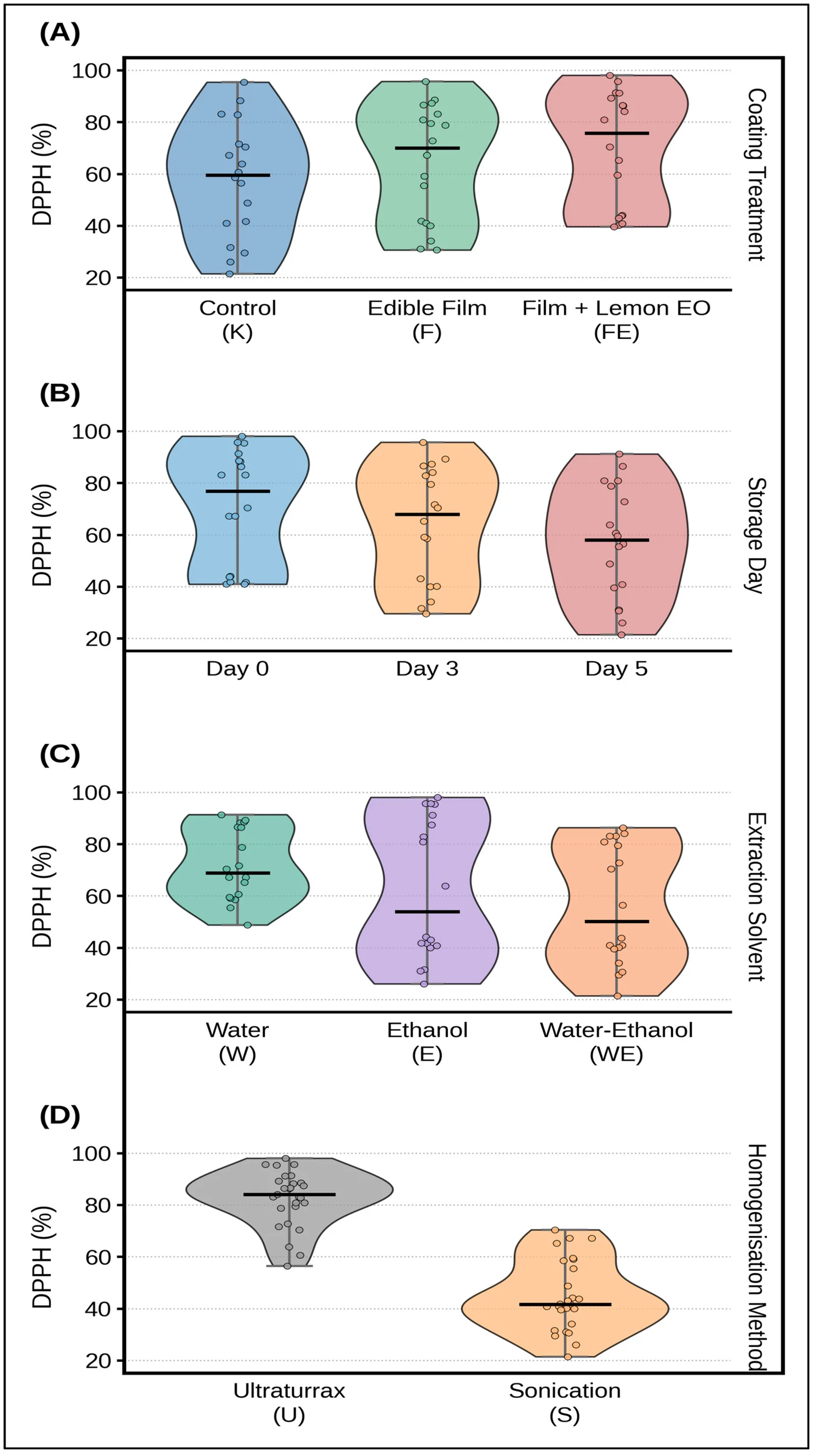
Violin plots illustrating the distribution of DPPH radical scavenging activity (%) in strawberry extracts depending on coating treatment (**A**), storage day (**B**), extraction solvent (**C**), and homogenization method (**D**). K, uncoated fruits as control; F, coated fruits with SA:G; FE, coated fruits with SA:G:LEO. Pooled values in panels (**C**,**D**) reflect variability across experimental combinations rather than independent replicate error (Section 2.13).

DPPH radical scavenging activity ranged from 21.38% to 98.11% across experimental conditions, showing that it depended on both coating treatment and extraction method (Table S5a–c). The DPPH radical scavenging activity values recorded at day 0 in the control group are consistent with the 20–95% inhibition range commonly reported in the literature for strawberry extracts prepared under varying solvent and homogenization conditions [39,40]. As illustrated in Figure 2A, the violin distributions clearly differentiate the three coating groups: (i) the SA:G:LEO (FE) treatment retained the highest median DPPH values with the narrowest distributional spread, (ii) F occupied an intermediate position, and (iii) K exhibited the lowest median with the broadest downward distribution. DPPH activity was lower in the absence of essential oil enrichment, although part of the higher activity in FE may come from the oil itself.

At day 0, coating groups did not differ (K: 69.46%, F: 69.54%, FE: 72.36%; *p* > 0.05), whereas differences among coatings were detected by days 3 and 5 (*p* < 0.05; exploratory). The treatment-dependent divergence that emerged during storage (Figure 2B) was clear by day 5: DPPH activity in the control had declined by 33.5%, while F and FE coatings restricted losses to 16.3% and 8.2%, respectively.

To determine whether the additional activity in FE-coated fruit came from the coating matrix or from the essential oil, the blank SA:G solution and LEO were each analyzed independently of the fruit. The blank solution showed no detectable radical scavenging activity (0%), so the polysaccharide–protein matrix alone contributes no measurable antioxidant capacity. LEO, in contrast, was active under all six extraction conditions (27.7–35.6%, mean 31.7%), which shows that the essential oil, not the matrix, can contribute radical-scavenging activity. This agrees with the radical-scavenging activity reported for limonene, the principal constituent of the lemon essential oil used here (72.85%) [41]. Because the oil itself scavenges DPPH radicals, part of the higher activity in FE extracts may come from oil co-extracted from the fruit surface rather than from better-preserved fruit antioxidants. The progressive downward shift in the day 5 violin body relative to day 0 (Figure 2B) illustrates the time-dependent oxidative deterioration, which was most pronounced in uncoated tissue.

Extraction solvent polarity exerted a substantial influence on measured DPPH values (Figure 2C). Aqueous extraction yielded the highest activity (71.84 ± 13.77%; the SD reflects variability across experimental combinations rather than replicate error, Section 2.13) and the most compact distribution. This is consistent with the predominantly hydrophilic character of strawberry antioxidants, notably anthocyanins, ascorbic acid, and water-soluble phenolic glycosides. Ethanol extraction produced a broader spread, which may reflect more variable recovery of moderately polar compounds such as hydroxycinnamic acids and flavonols. On the other hand, the water–ethanol binary system yielded the lowest mean values (56.53 ± 23.02%). The individual phenolic data showed the same pattern: water–ethanol extracts had the lowest mean concentration for all 14 compounds (Table S7a).

The homogenization method emerged as the most discriminating pre-analytical variable across the entire dataset (Figure 2D). Ultra-Turrax-assisted disruption produced a mean DPPH activity of 82.58 ± 10.75%, nearly double that obtained by sonication (44.88 ± 13.56%). The clear separation between the two violin distributions, with virtually no overlap in their core ranges, indicates that acoustic cavitation was considerably less effective than high-shear mechanical disruption under the applied conditions. This lower efficiency may reflect a reduced capacity to break down the polysaccharide-rich cell wall matrix of strawberry parenchyma and release its intracellular antioxidant content.

Overall, the FE coating was associated with the smallest loss of DPPH activity during storage at room temperature. The blank solution had no DPPH activity, so the matrix alone does not explain this difference; part of it may reflect the oil itself rather than better-preserved fruit antioxidants. The extraction protocol, particularly the homogenization method, strongly affects the measured antioxidant activity and should be standardized across studies to allow comparison.

### 3.6. Total Phenolic Content

Total phenolic content (TPC) ranged from 106.54 to 485.45 mg GAE/100 g fw across all experimental conditions (Table S6a–c). The highest TPC was recorded in FE group strawberries extracted with ethanol under Ultra-Turrax homogenization at day 0, and the lowest was found in uncoated control samples extracted with the water–ethanol mixture by sonication at day 5. Previous reports give TPC values of 210–775 mg GAE/100 g fw, and TPC may vary with cultivar, extraction solvent, and homogenization method [42,43]. The initial TPC values of the control group in the present study fell within this range.

**Figure 3:**
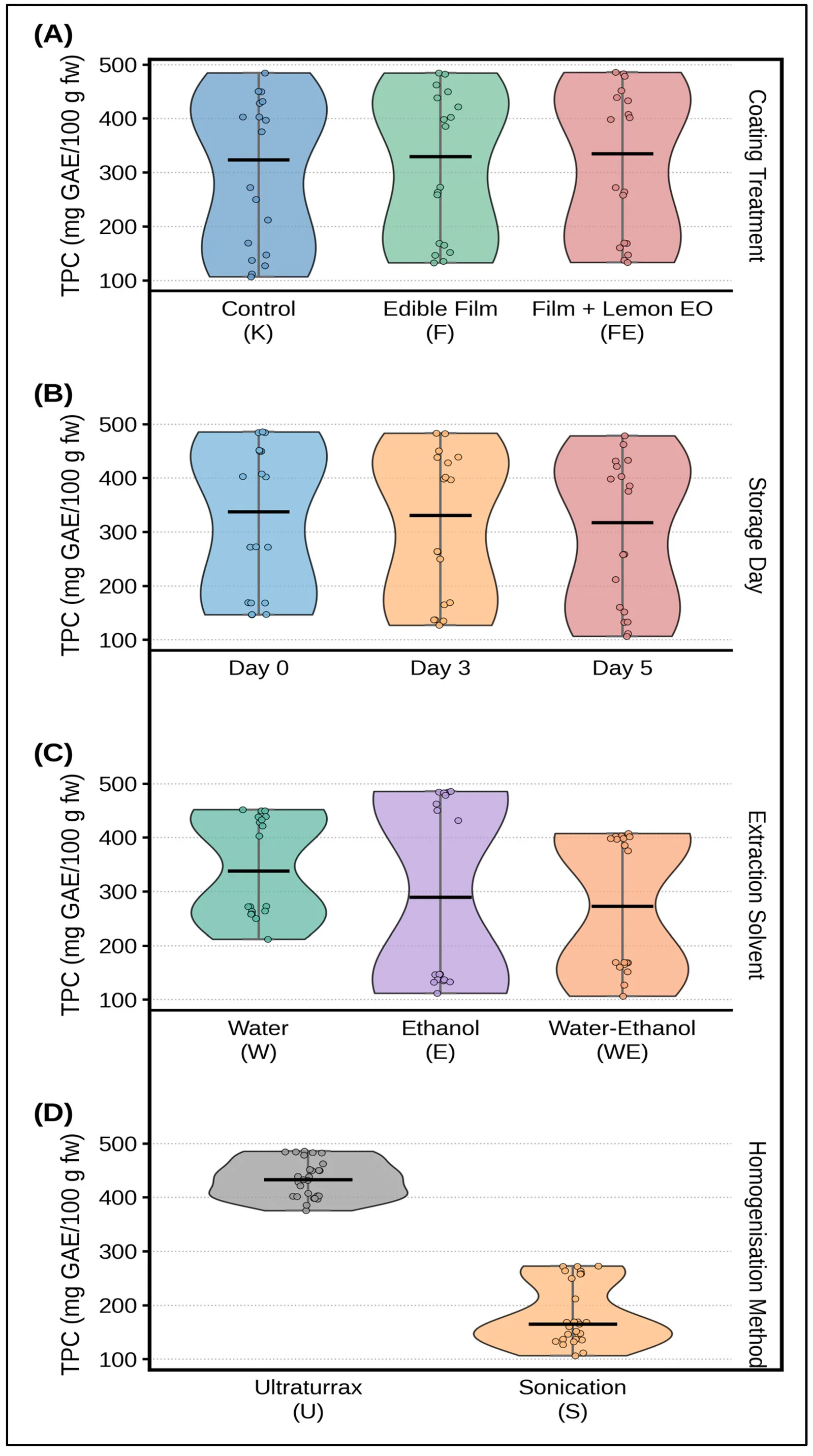
Violin plots illustrating the distribution of total phenolic content (mg GAE/100 g fw) in strawberry extracts depending on coating treatment (**A**), storage day (**B**), extraction solvent (**C**) and homogenization method (**D**). (K, uncoated fruits as control; F, coated fruits with SA:G; FE, coated fruits with SA:G:LEO). Pooled values in panels (**C**,**D**) reflect variability across experimental combinations rather than independent replicate error (Section 2.13).

As shown in Figure 3A, all trial groups exhibited virtually identical mean TPC values at day 0 (K: 320.73, F: 320.74, FE: 322.09 mg GAE/100 g fw), and the groups did not differ immediately after harvest (*p* > 0.05). TPC differed among coating treatments from day 3 onward (*p* < 0.05; exploratory) (Figure 3B). By day 5, TPC in the control group had declined by 14.7%, whereas F and FE coatings restricted losses to 5.8% and 3.7%, respectively. In the control group the loss accelerated toward the end of storage: and a further 8.3% between days 3 and 5 (relative to the day-3 value). Phenolic losses of this kind have been linked to senescence-related membrane damage that exposes substrates to polyphenol oxidase, a process that speeds up at ambient temperature [38]. Microbial activity may also contribute, which would agree with the higher yeast and mold counts recorded in the control group (Section 3.3). Retention of total phenolics by edible coatings under ambient storage has also been reported for strawberries [35].

The blank SA:G solution and LEO were also analyzed independently of the fruit. The blank solution showed no reactivity in the Folin–Ciocalteu assay, whereas LEO gave a measurable signal under all six extraction conditions (15.33–28.82 mg GAE/g), the highest with water–ethanol and Ultra-Turrax (28.82 mg GAE/g) and the lowest with ethanol and sonication (15.33 mg GAE/g). The coating matrix therefore does not contribute to the measured TPC, but the essential oil does. None of the 14 individual phenolics quantified by HPLC were detected in LEO, so its TPC signal arises from compounds outside that panel. Part of the higher TPC in FE-coated fruit could thus come from LEO itself, co-extracted from the fruit surface, rather than from better-preserved fruit phenolics; the individual phenolic profile (Section 3.7) gives a cleaner measure of fruit phenolic retention.

Solvent polarity and homogenization method had considerable effects on extractable TPC (Figure 3C,D). Among the three solvent systems, water extraction yielded the highest values and the most compact distribution (346.45 ± 92.58 mg GAE/100 g fw; SD reflecting structural variability across experimental combinations rather than replicate error, as described in Section 2.13). Ethanol showed the widest distribution range, which may reflect its more variable recovery of moderately polar phenolic compounds. The water–ethanol mixture yielded the lowest mean TPC (275.26 ± 125.94 mg GAE/100 g fw). The phenolic yield obtained through Ultra-Turrax homogenization (434.28 ± 34.50 mg GAE/100 g fw) was more than twice that obtained by sonication (182.82 ± 57.35 mg GAE/100 g fw), with virtually non-overlapping violin distributions (Figure 3D). This near-complete separation indicates that the efficiency of cell wall disruption is a primary determinant of the extractable phenolic yield in strawberry tissue, and that methodological standardization is important for valid inter-study comparisons in fruit phenolic research.

### 3.7. Phenolic Diversity

A total of 14 individual phenolic compounds were identified and quantified in strawberry samples (Table S7a,b), including flavan-3-ols (catechin, epicatechin, epigallocatechin), procyanidins (procyanidin B1 and B2), phenolic acids (gallic, ferulic, chlorogenic, caffeic, neochlorogenic and *p*-coumaric acid) and flavonols (quercetin-3-glucoside, kaempferol-3-glucoside and rutin). In the uncoated control group at day 0, the dominant phenolic compound was catechin (4.49–10.49 mg/100 g fw). Among phenolic acids, gallic acid was present in the highest concentrations (2.22–3.56 mg/100 g fw), followed by *p*-coumaric acid (1.45–1.81 mg/100 g fw), chlorogenic acid (1.11–1.58 mg/100 g fw) and ferulic acid (0.49–1.12 mg/100 g fw). In the flavonol group, quercetin-3-glucoside was the most abundant (1.00–1.17 mg/100 g fw), followed by kaempferol-3-glucoside (0.23–0.89 mg/100 g fw) and rutin (0.31–0.71 mg/100 g fw). These values are in line with earlier reports [44,45,46,47].

The blank SA:G solution and LEO were also screened for the same 14 phenolic compounds. None of the compounds were detected in either the blank solution or LEO under any of the six extraction conditions. This indicates that the coating matrix and the essential oil do not themselves contribute to the individual phenolic concentrations measured in the coated fruit; the phenolics quantified in F- and FE-coated samples therefore originate from the fruit itself, and any protective effect of the coating on these compounds reflects preservation of the fruit’s own phenolic content rather than an additive contribution from the coating materials.

All 14 phenolic compounds differed among coating treatments by day 5 (*p* < 0.05; exploratory). Uncoated control samples showed a marked decrease in all 14 compounds, whereas coated fruits retained more of their individual phenolics during storage. Using the water–Ultra-Turrax system as the reference extraction protocol, catechin declined by 88.9% in the control by day 5, while F and FE coatings limited this loss to 2.8% and 2.2%, respectively. Other phenolics showed similar differences: epicatechin (96.2% in the control vs. 27.9% in F and 20.1% in FE), procyanidin B1 (97.4%, 8.7% and 3.4%), *p*-coumaric acid (99.7% vs. 15.3% and 9.6%), kaempferol-3-glucoside (99.6% vs. 15.7% and 12.7%) and rutin (98.9% vs. 9.4% and 10.0%). The large loss of phenolic compounds in the control group is consistent with polyphenol oxidase-driven enzymatic browning, microbial activity, and non-enzymatic oxidative polymerization at ambient temperature, although we did not measure these mechanisms individually. A physical barrier effect of the coating may explain part of the higher retention in coated fruit, and the additional retention in FE-coated fruit may be related to the antioxidant and antimicrobial properties of LEO. We did not measure oxygen permeability, enzyme activity, or the interaction between LEO and fruit tissue, so these explanations are possible interpretations and were not tested. Three points should be considered when reading these losses. First, the same extraction and HPLC procedures were applied to all coating groups, and coated fruit lost only 2.5–29.7% of the same compounds, so a lower extraction efficiency or an instrument problem alone cannot explain the difference between the control and coated fruit. Mean day-0 concentrations across extraction conditions were comparable among coating groups. Second, concentrations in the uncoated control decreased in all six solvent–homogenization combinations for every compound (Table S7a), which argues against a single extraction condition driving the result. Third, the day-5 concentrations in the control were very low for several compounds, so the percentage losses given for the control are approximate and should be read as a large decrease rather than as exact values.

The influence of extraction conditions on phenolic diversity and content was also examined. Both solvent type and homogenization technique influenced the phenolic profile (*p* < 0.05; exploratory), consistent with previous studies addressing phenolic extraction from plant matrices [48,49]. Quantitative differences depended on the polarity of the compound in question. In the uncoated control at day 0, for example, epicatechin reached higher concentrations with ethanol (0.97–1.00 mg/100 g fw) than with water (0.67–0.74 mg/100 g fw), a pattern that has been attributed to the relatively hydrophobic character of epicatechin [50].

**Figure 4:**
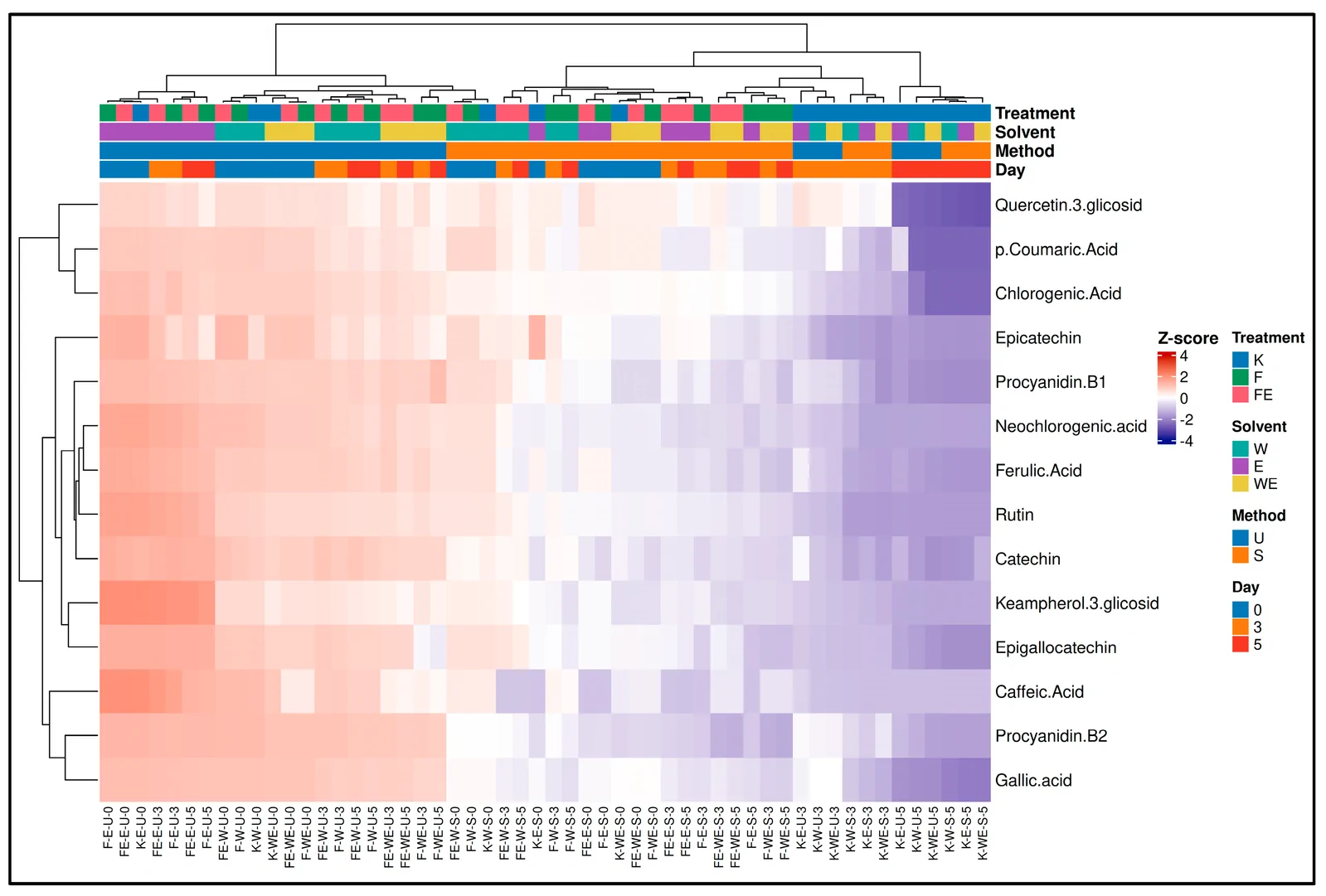
Hierarchical clustering heatmap of individual phenolic compound profiles (14 compounds) in strawberry extracts (n = 54). Heatmap values are z-score normalized; color scale ranges from deep blue (z = −4) to deep red (z = +4). Annotation bars indicate coating treatment, storage day, extraction solvent and homogenization method.

Hierarchical clustering heatmap analysis was performed to visualize the combined effects of coating treatment, storage day, extraction solvent and homogenization method on the phenolics in strawberry extracts (Figure 4). The most depleted sample across the panel was the uncoated control extracted with water–ethanol by sonication at day 5 (K-WE-S-5), which held the lowest concentration for 13 of the 14 compounds. The absence of a coating, the lower recovery typical of sonication, and prolonged storage are the most plausible contributors to this pattern. The highest concentrations were generally found in F and FE samples extracted with ethanol under Ultra-Turrax homogenization at day 0. Averaged across all storage days and solvents, catechin reached a mean of 9.60–9.70 mg/100 g fw in Ultra-Turrax-extracted F and FE samples, compared with a mean of 4.84 mg/100 g fw in the control. Gallic acid, rutin, *p*-coumaric acid and quercetin-3-glucoside followed a similar pattern. In Figure 4, this appears as a consistent red block over the F and FE columns at days 0–3, and a deep blue block over the control columns at day 5. The heatmap also showed a three-part structure corresponding to the three storage days, with storage day representing the dominant source of variation across the dataset. Control samples at day 5 clustered tightly with uniformly deep blue z-scores, reflecting near-total phenolic loss, while day 0 samples across all treatment groups occupied the deep red region and day 3 samples fell in between. F and FE samples at day 5 retained z-scores close to their day 0–3 profiles, illustrating the protective effect of both formulations. Within the compound axis, flavan-3-ols and procyanidins clustered together as the most labile fraction, while flavonols and hydroxycinnamic acids formed a separate group.

**Figure 5:**
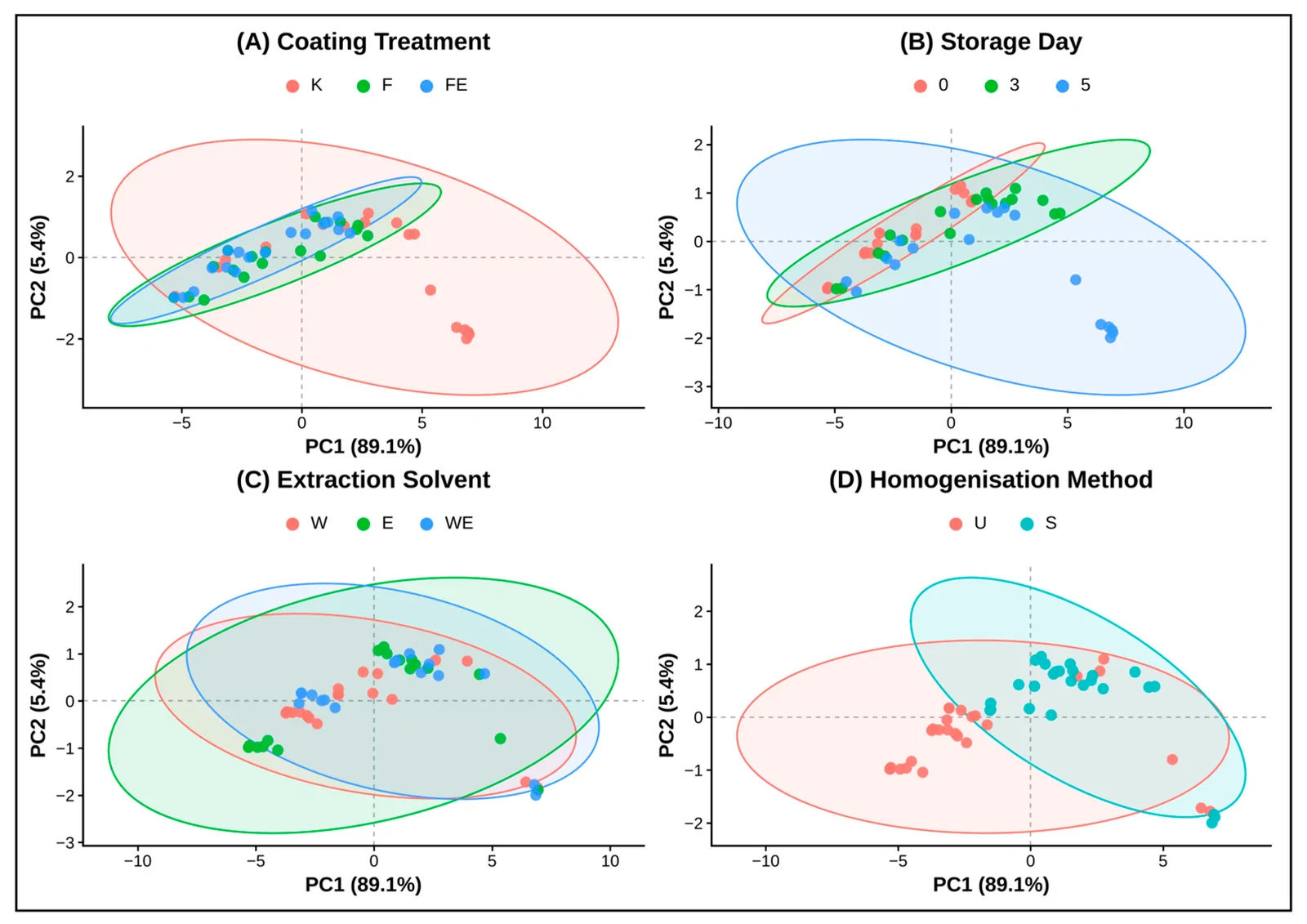
Principal component analysis (PCA) scores plots of individual phenolic compound profiles (14 compounds) in strawberry extracts (n = 54), colored by coating treatment (**A**), storage day (**B**), extraction solvent (**C**), and homogenization method (**D**). Each point is one factorial combination (one measured value). Group sizes are n = 18 for coating treatment, storage day, and extraction solvent, and n = 27 for homogenization method. Ellipses are descriptive 95% normal-theory ellipses for each group; loading vectors indicate the five variables with the greatest contribution to PC1. (K, uncoated fruits as control; F, coated fruits with SA:G; FE, coated fruits with SA:G:LEO).

Principal component analysis was performed on the 14-compound phenolic dataset to examine how coating treatment, storage day, extraction solvent and homogenization method shape the overall phenolic profile (Figure 5). PC1 and PC2 explained 89.1% and 5.4% of the total variance, respectively (94.5% combined), indicating that phenolic variation across experimental conditions is governed almost entirely by a single dominant axis. All phenolics loaded in the same direction along PC1, reflecting a shared response to storage-related degradation. Panel B showed a clear separation by storage day: control samples at day 5 projected to strongly negative PC1 scores, while coated samples at days 0 and 5 clustered at the positive end, with little overlap between the two.

This separation indicates that the coatings substantially limited the phenolic loss that occurred in untreated fruit. Panel (A) showed that F and FE were indistinguishable at day 0 but diverged progressively during storage, with FE samples at day 5 reaching slightly higher PC1 scores than F, a small difference that we do not interpret further. In panel C, ethanol-extracted samples formed a relatively distinct group, whereas water and water–ethanol extracts formed broader, overlapping clusters. Homogenization method (panel D) produced the clearest separation among the four factors: Ultra-Turrax- and sonication-extracted samples formed two largely non-overlapping clusters along PC1, indicating that mechanical disruption strongly affects the recovery of phenolic compounds from strawberry tissue. Because each factorial combination is represented by a single measured value, these score plots describe the structure of the 54 combinations rather than sampling variability, and small clusters are not interpreted individually.

### 3.8. SVR Modelling

SVR with an RBF kernel was used to model the concentrations of 14 individual phenolic compounds as a function of four experimental factors: coating treatment, extraction solvent, homogenization method and storage day. Hyperparameter selection and performance evaluation were separated using nested cross-validation to obtain a less optimistic estimate of generalization (Section 2.12); results are summarized in Table 1. Of the 14 compounds, 8 reached a nested cross-validated R^2^ ≥ 0.85, 4 fell in the 0.70–0.85 range, and 2 remained below 0.70. The highest performance was recorded for ferulic acid (R^2^ = 0.952 ± 0.023), chlorogenic acid (R^2^ = 0.950 ± 0.020), and catechin (R^2^ = 0.934 ± 0.071). For most compounds, the nested cross-validated R^2^ was noticeably lower than the R^2^ obtained by fitting and evaluating the same model on the complete dataset, with a mean gap of 0.17 across all 14 compounds (Table S8). This gap indicates some overfitting, which is expected given the small sample size (n = 54) relative to the hyperparameter search space; the nested cross-validated values, not the full-dataset fit, are used throughout as the performance estimate. With only 54 combinations and no independent validation set, these values indicate how well the models fit this dataset and should not be read as the accuracy expected on new samples.

Performance was lowest for *p*-coumaric acid (R^2^ = 0.619 ± 0.337) and quercetin-3-glucoside (R^2^ = 0.366 ± 0.116), both of which also showed the largest train–test gaps among the 14 compounds (0.30 and 0.63, respectively; Table S8), and *p*-coumaric acid also showed the highest fold-to-fold variability, indicating that the models fit these two compounds well on the full dataset but did not generalize to held-out samples. A plausible biological contributor is their instability during storage: *p*-coumaric acid is a phenylpropanoid pathway intermediate subject to rapid polyphenol oxidase and peroxidase activity, with concentrations in most uncoated control samples falling to very low levels by day 5 (Section 3.7), while quercetin-3-glucoside’s stability has been linked to concurrent ascorbic acid oxidation and anthocyanin degradation [40,50,51]. Neither ascorbic acid oxidation state nor anthocyanin content was quantified here, so this mechanism is inferred rather than demonstrated. This explanation cannot be separated from a simpler one: with only 54 samples split across five outer folds (roughly 10–11 observations per fold), a handful of atypical values for a low-concentration, fast-degrading compound could disproportionately affect both model fitting and evaluation. The present data cannot distinguish a genuinely irregular biological trajectory from a sample size too small to characterize it reliably.

**Figure 6:**
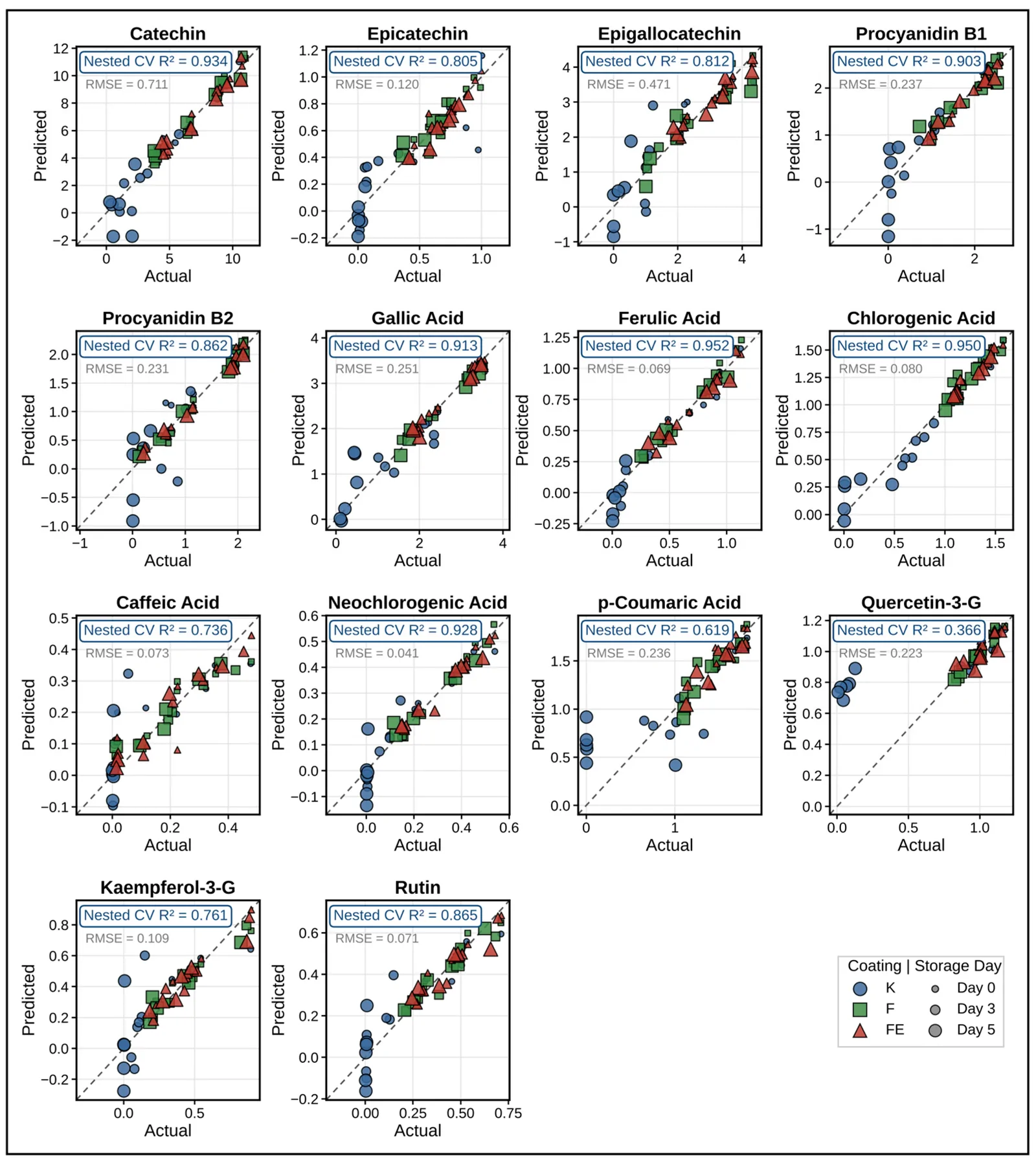
Observed versus predicted scatter plots for the SVR–RBF models of all 14 individual phenolic compounds, based on out-of-fold predictions from nested cross-validation. The dashed line represents the ideal 1:1 fit. Point color and shape correspond to coating treatment; point size corresponds to storage day. (K, uncoated fruits as control; F, coated fruits with SA:G; FE, coated fruits with SA:G:LEO).

**Figure 7:**
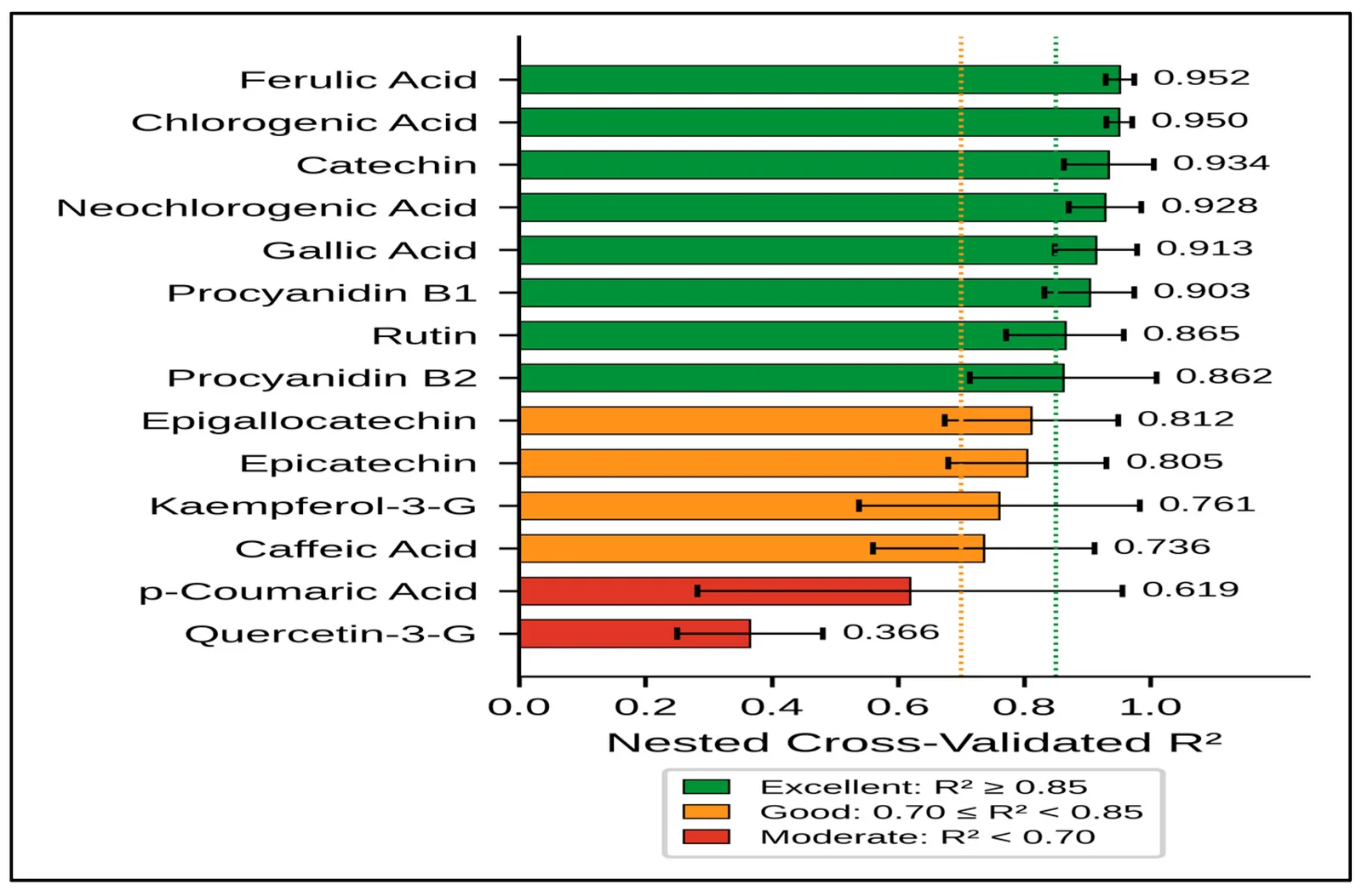
Nested cross-validated R^2^ performance chart for the SVR–RBF models of all 14 individual phenolic compounds, grouped into three performance tiers: excellent (R^2^ ≥ 0.85), good (0.70 ≤ R^2^ < 0.85) and moderate (R^2^ < 0.70).

The observed-versus-predicted comparisons in Figure 6 use out-of-fold predictions, so each point reflects a model that had not seen that sample during training or hyperparameter selection. Data points clustered closely around the 1:1 line for most compounds, with tighter clustering for higher-R^2^ compounds and a visibly broader spread for quercetin-3-glucoside and *p*-coumaric acid, reflecting larger prediction errors. For several low-concentration compounds (catechin, procyanidin B1, procyanidin B2, ferulic acid, kaempferol-3-glucoside, rutin), a few near-zero observed values were paired with slightly negative predictions, an expected behavior of an unconstrained regression model. The R^2^ performance chart (Figure 7) summarizes these results across three tiers: excellent (R^2^ ≥ 0.85: ferulic acid, chlorogenic acid, catechin, neochlorogenic acid, gallic acid, procyanidin B1, rutin, procyanidin B2), good (0.70–0.85: epigallocatechin, epicatechin, kaempferol-3-glucoside, caffeic acid), and moderate (<0.70: *p*-coumaric acid, quercetin-3-glucoside).

**Figure 8:**
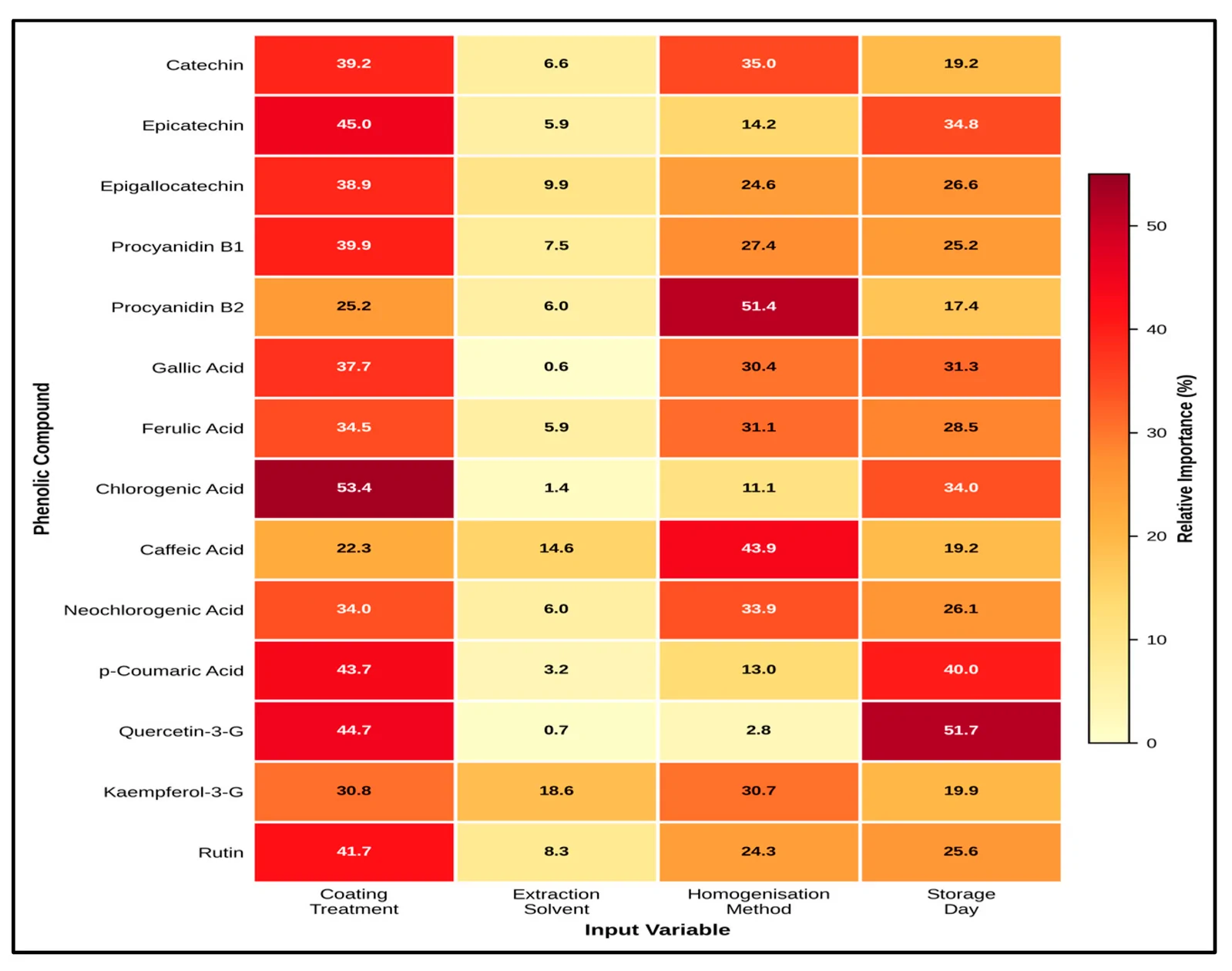
Permutation-based feature importance for the SVR–RBF models of individual phenolic compounds, showing the relative contribution of coating treatment, storage day, extraction solvent and homogenization method to model predictions.

The final model used for this analysis was fit on the complete dataset for each compound, with hyperparameters selected via the same grid search procedure described in Section 2.12 (Table S8); these differ from the fold-specific hyperparameters underlying the nested cross-validated performance in Table 1. Permutation-based feature importance (Figure 8) identified coating treatment as the most influential predictor overall (mean relative importance 37.9%), followed by storage day (28.5%) and homogenization method (26.7%), with extraction solvent contributing least (6.8%). This ranking varied by compound: homogenization method dominated for procyanidin B2 and caffeic acid (51.4% and 43.9%), while storage day and coating treatment dominated for quercetin-3-glucoside and chlorogenic acid (51.7% and 53.4%), indicating that no single factor governs all 14 phenolics uniformly. Two of the four input variables, coating treatment and storage day, describe the biological history of the fruit, while the other two, extraction solvent and homogenization method, describe how each sample was subsequently analyzed. The models therefore describe the phenolic concentration as measured under a given extraction protocol, not an underlying in vivo concentration independent of that protocol; the two cannot be separated with the present design, since every sample was extracted under one specific solvent–method combination rather than multiple methods applied to the same tissue. Overall, the nested cross-validated results show that these four factors together captured a substantial share of the variability in most individual phenolics, with 12 of 14 compounds reaching R^2^ ≥ 0.70; performance was distinctly lower for *p*-coumaric acid and quercetin-3-glucoside, for the reasons discussed above. These models describe patterns within the present, relatively small dataset (n = 54) and have not been tested against independent samples; they are presented as an exploratory characterization of how these biological and analytical factors jointly relate to the measured phenolic concentration, not as a validated predictive tool.

## 4. Conclusions

This study showed that a LEO-enriched alginate–gelatin coating (FE) limits weight loss, microbial growth, and the loss of color and individual phenolics in strawberries stored at ambient temperature, and that the extraction protocol used to measure these compounds affects the measured values to an extent comparable to the coating treatment. The FE coating limited weight loss to 18.94% and yeast and mold counts to 1.10 log CFU/g by day 5, compared with 28.99% and 5.33 log CFU/g in the uncoated control; *Escherichia coli* was not detected in any sample. FE-coated fruit also retained color (L*, a*, b*) closer to its initial values throughout storage. Biochemically, FE treatment limited DPPH and TPC losses to 8.2% and 3.7%, respectively, and substantially reduced the loss of individual phenolics relative to the uncoated control, where several compounds almost disappeared by day 5 (in the water–Ultra-Turrax system, losses were 98.9% for rutin, 99.6% for kaempferol-3-glucoside, and 99.7% for *p*-coumaric acid). The F coating without LEO gave similar phenolic retention, so most of this protection can be attributed to the alginate–gelatin matrix; LEO had a clearer additional effect on yeast and mold counts. Analysis of the blank coating solution and LEO independently of the fruit showed that the blank solution had no DPPH or Folin–Ciocalteu activity while LEO had both. The higher DPPH and TPC values of FE-coated fruit may therefore partly reflect co-extracted oil, and the individual phenolic data are the cleaner evidence of fruit phenolic retention. The homogenization method was the more decisive extraction variable for antioxidant activity and total phenolic content, with Ultra-Turrax consistently outperforming sonication, indicating that extraction protocols should be reported and standardized in strawberry phenolic research. These findings should be interpreted with some limitations in mind: anthocyanin content was not measured, despite its relevance to several of the degradation mechanisms discussed. Barrier properties (water vapor and oxygen transmission rates) and fruit respiration rate were not measured, so the mechanisms proposed for the effects on weight loss and microbial growth remain untested. Reported SD values for pooled extraction-protocol comparisons (by solvent or homogenization method) reflect structural variability across experimental combinations rather than independent replicate error, as detailed in Section 2.13.

An SVR model relating coating treatment, extraction solvent, homogenization method, and storage day to the concentration of each individual phenolic, evaluated by nested cross-validation, reached R^2^ ≥ 0.85 for 8 of the 14 compounds and R^2^ ≥ 0.70 for 12 of the 14; performance was distinctly lower for *p*-coumaric acid and quercetin-3-glucoside. Because two of the four input variables describe the fruit’s biological history and the other two describe how each sample was subsequently analyzed, the model characterizes the measured phenolic concentration under a given extraction protocol rather than an underlying in vivo value independent of it. Permutation-based feature importance identified coating treatment as the most influential factor overall, followed by storage day and homogenization method, with extraction solvent contributing least, though this ranking varied by compound. These models were not tested against independent samples and are presented as an exploratory characterization of these relationships rather than a validated predictive tool; extending them to new formulations or storage conditions would require testing on data collected under those conditions. Both the extraction protocol used to assess phenolic quality and the modeling approach applied to it warrant further attention in postharvest phenolic research.

## Figures and Tables

**Table 1:** Nested cross-validated performance of the SVR–RBF models for individual phenolic compounds.

Target	Nested CV R^2^	RMSE	MAE
Ferulic Acid	0.952 ± 0.023	0.069	0.055
Chlorogenic Acid	0.950 ± 0.020	0.080	0.057
Catechin	0.934 ± 0.071	0.711	0.542
Neochlorogenic Acid	0.928 ± 0.058	0.041	0.030
Gallic Acid	0.913 ± 0.065	0.251	0.168
Procyanidin B1	0.903 ± 0.071	0.237	0.169
Rutin	0.865 ± 0.093	0.071	0.054
Procyanidin B2	0.862 ± 0.147	0.231	0.165
Epigallocatechin	0.812 ± 0.137	0.471	0.361
Epicatechin	0.805 ± 0.126	0.120	0.085
Kaempferol-3-glucoside	0.761 ± 0.222	0.109	0.078
Caffeic Acid	0.736 ± 0.176	0.073	0.054
*p*-Coumaric Acid	0.619 ± 0.337	0.236	0.157
Quercetin-3-glucoside	0.366 ± 0.116	0.223	0.110

Values are the mean and standard deviation of R^2^, RMSE, and MAE across five outer test folds of a nested cross-validation scheme, in which hyperparameter selection (inner five-fold grid search) and performance evaluation (outer fold) never used the same data. Hyperparameters were selected independently within each outer fold and are therefore not reported as a single fixed set. RMSE and MAE are expressed in mg/100 g fw.

## Data Availability

The original contributions presented in this study are included in this article/Supplementary Material. Further inquiries can be directed to the corresponding author.

## Supplementary Materials

The following supporting information can be downloaded at: https://www.mdpi.com/article/10.3390/foods15213834/s1, Table S1a. Weight loss (%) by coating treatment and storage day (mean ± SD, n = 18 per cell). Table S1b. Two-way ANOVA for weight loss (coating treatment × storage day). Not: Mean and SD reflect n = 18 fruit (three replicates × six fruit per replicate; Section 2.5). F and *p* values were reconstructed from the cell means and SDs. Table S2a. Yeast and mold counts (log CFU/g) by coating treatment and storage day (mean ± SD). Table S2b. Two-way ANOVA for yeast and mold counts (coating treatment × storage day). Not: F and *p* values were reconstructed from the cell means and SDs. Table S3. *E. coli* counts (log CFU/g) by coating treatment and storage day. Neither coliforms nor E. coli were detected in any of the 27 samples (K/F/FE × day 0/3/5 × 3 replicates). No statistical comparison was made, because all values were identical. Table S4a. Fruit color (L*, a*, b*) by coating treatment and storage day (mean ± SD, n = 4 per cell). Table S4b–d. Two-way ANOVA for L* (b), a* (c) and b* (d) (coating treatment × storage day). Not: ANOVA was calculated from the replicate-level color measurements (Section 2.6). Table S5a. DPPH radical scavenging activity (%) in the full 54-sample factorial matrix. Table S5b. DPPH radical scavenging activity pooled by factor (treatment, storage day, solvent, homogenization method; mean ± SD, n = 18 per level). Table S5c. Four-way factorial ANOVA for DPPH (treatment × solvent × method × day, with two-way interactions). Table S6a. Total phenolic content (mg GAE/100 g fw) in the full 54-sample factorial matrix. Table S6b. TPC pooled by factor (mean ± SD, n = 18 per level). Table S6c. Four-way factorial ANOVA for TPC (treatment × solvent × method × day, with two-way interactions). Table S7a. Concentrations of the 14 individual phenolic compounds (mg/100 g fw) in the full 54 × 14 factorial matrix. Table S7b. Per-compound ANOVA (main effects of treatment, solvent, method and day; n = 54 per compound). Not: EGC, epigallocatechin; PB1/PB2, procyanidin B1/B2; Q-3-gluc, quercetin-3-glucoside; K-3-gluc, kaempferol-3-glucoside. Table S8. Hyperparameters, and train and nested cross-validated performance, of the final SVR–RBF model fit on the complete dataset (n = 54) for each phenolic compound. These models were used only for the permutation-based feature importance analysis (Figure 8). They differ from the fold-specific hyperparameters selected within each outer fold of the nested cross-validation (Section 2.12), which underlie the performance metrics in Table 1.

## Abbreviations

The following abbreviations are used in this manuscript:

ANOVA	Analysis of Variance
CV	Cross-Validation
DPPH	2,2-diphenyl-1-picrylhydrazyl
F	Edible film (SA:G coating without LEO)
FE	LEO-enriched edible film (SA:G:LEO coating)
G	Gelatin
GAE	Gallic Acid Equivalents
GC-MS	Gas Chromatography–Mass Spectrometry
HPLC	High-Performance Liquid Chromatography
K	Uncoated Control
LEO	Lemon Essential Oil
MAE	Mean Absolute Error
PCA	Principal Component Analysis
RBF	Radial Basis Function
RMSE	Root Mean Square Error
SA	Sodium Alginate
SVR	Support Vector Regression
TPC	Total Phenolic Content
