# Supplementary material for: Lemon Essential Oil-Enriched Alginate–Gelatin Coating Preserves Bioactive Quality in Strawberries: Extraction Protocol Effects and an Exploratory Modeling Approach
Source: Foods. 2026 Oct 27;15(21):3834. doi: 10.3390/foods15213834 (PMC13763031; doi:10.3390/foods15213834)
Supplement: Supplementary file 1 [file foods-15-03834-s001.zip › foods-4635187-supplementary.pdf]

Supplementary Materials

Tables S1–S8 report full numerical means, SDs, n values, and ANOVA p-values for weight loss, color, microbial counts, DPPH, TPC, and individual phenolics across all treatment × day × solvent × homogenization combinations. No new experiments were performed; all values are derived from the dataset underlying the main manuscript.

Table S1a. Weight loss (%) by coating treatment and storage day (mean ± SD, n = 18 per cell).

Treatment	Day	Mean (%)	SD	n
K	0	0	0	18
K	3	16.35	0.01	18
K	5	28.99	0.02	18
F	0	0	0	18
F	3	14.23	0	18
F	5	20.16	0.01	18
FE	0	0	0	18
FE	3	15.35	0.01	18
FE	5	18.94	0.01	18

Table S1b. Two-way ANOVA

Source	F	df1	df2	p
Treatment	2722668.75	2	153	<0.001
Day	81414884.25	2	153	<0.001
Treatment*Day	1797810.75	4	153	<0.001

K, uncoated fruit (control); F, coated fruit with SA:G; FE, coated fruit with SA:G:LEO. Mean and SD reflect n = 18 independent fruit (three replicates × six fruit per replicate), as described in Section 2.4. F- and p-values were reconstructed from these cell-level means and SDs.

Table S2a. Yeast/mold counts (log CFU/g) by coating treatment and storage day (mean ± SD, n = 18 per cell).

Treatment	Day	Mean (log CFU/g)	SD	n
K	0	1.7	0.008	18
K	3	3.67	0.002	18
K	5	5.33	0.001	18
F	0	0.67	0.001	18
F	3	2.03	0.012	18
F	5	2.83	0.011	18
FE	0	0	0	18
FE	3	0	0	18
FE	5	1.1	0.001	18

Table S2b. Two-way ANOVA

Source	F	df1	df2	p
Treatment	3710191.07	2	153	<0.001
Day	1908069.64	2	153	<0.001
Treatment*Day	226757.14	4	153	<0.001

K, uncoated fruit (control); F, coated fruit with SA:G; FE, coated fruit with SA:G:LEO. The same n = 18 sampling scheme reported for weight loss (Section 2.4) was assumed here, as the Methods do not separately state a replicate number for the microbiological analysis.

Table S3. *E. coli* counts (log CFU/g) by coating treatment and storage day.

Treatment	Day	Mean	SD	n
F	0	0	0	3
F	3	0	0	3
F	5	0	0	3
FE	0	0	0	3
FE	3	0	0	3
FE	5	0	0	3
K	0	0	0	3
K	3	0	0	3
K	5	0	0	3

All 27 samples (K/F/FE × day 0/3/5 × 3 replicates) were below the detection limit; no statistical comparison was performed given zero variance across all groups.

Table S4a. Fruit color (L*, a*, b*) by coating treatment and storage day (mean ± SD, n = 4 per cell).

Treatment	Day	n	L* Mean	L* SD	a* Mean	a* SD	b* Mean	b* SD
F	0	4	34.425	1.286	25.75	1.554	17.450	0.299
F	3	4	28.724	0.464	22.325	0.262	15.4	0.294
F	5	4	27.075	0.309	18.275	0.386	14.5	0.270
FE	0	4	35.300	0.867	25.575	0.262	17.625	0.287
FE	3	4	29.449	0.519	23.5	0.432	15.5	0.163
FE	5	4	28.200	0.668	19.475	0.170	14.725	0.095
K	0	4	35.8	0.816	25.825	0.670	17.05	0.613
K	3	4	25.85	0.718	21.375	1.007	13.524	0.464
K	5	4	23.175	0.974	16.725	0.309	11.425	0.590

Table S4b. ***Two-way ANOVA — L****

Source	SS	df	F	p
C(Treatment)	45.6	2	36.84	<0.001
C(Day)	545.06	2	440.35	<0.001
C(Treatment):C(Day)	42.91	4	17.33	<0.001

Table S4c. Two-way ANOVA — a*

Source	SS	df	F	p
C(Treatment)	14.27	2	14.33	<0.001
C(Day)	344.48	2	345.96	<0.001
C(Treatment):C(Day)	10.13	4	5.09	0.003

Table S4d. Two-way ANOVA — b*

Source	SS	df	F	p
C(Treatment)	28.04	2	96.33	<0.001
C(Day)	91.21	2	313.31	<0.001
C(Treatment):C(Day)	9.75	4	16.75	<0.001

K, uncoated fruit (control); F, coated fruit with SA:G; FE, coated fruit with SA:G:LEO. ANOVA computed directly from raw replicate-level color measurements (Section 2.5).

Table S5a. DPPH radical scavenging activity (%) — full 54-sample factorial matrix.

Sample code	Treatment	Solvent	Method	Day	DPPH (%)
K-W-U-0	K	W	U	0	88.288
K-W-S-0	K	W	S	0	67.181
K-E-U-0	K	E	U	0	95.447
K-E-S-0	K	E	S	0	41.660
K-WE-U-0	K	WE	U	0	83.178
K-WE-S-0	K	WE	S	0	40.985
F-W-U-0	F	W	U	0	88.526
F-W-S-0	F	W	S	0	67.213
F-E-U-0	F	E	U	0	95.734
F-E-S-0	F	E	S	0	41.758
F-WE-U-0	F	WE	U	0	83.149
F-WE-S-0	F	WE	S	0	40.886
FE-W-U-0	FE	W	U	0	91.437
FE-W-S-0	FE	W	S	0	70.449
FE-E-U-0	FE	E	U	0	98.111
FE-E-S-0	FE	E	S	0	44.065
FE-WE-U-0	FE	WE	U	0	86.406
FE-WE-S-0	FE	WE	S	0	43.689
K-W-U-3	K	W	U	3	71.604
K-W-S-3	K	W	S	3	58.557
K-E-U-3	K	E	U	3	82.842
K-E-S-3	K	E	S	3	31.490
K-WE-U-3	K	WE	U	3	70.436
K-WE-S-3	K	WE	S	3	29.509
F-W-U-3	F	W	U	3	86.615
F-W-S-3	F	W	S	3	59.113
F-E-U-3	F	E	U	3	87.397
F-E-S-3	F	E	S	3	39.948
F-WE-U-3	F	WE	U	3	79.436
F-WE-S-3	F	WE	S	3	34.016
FE-W-U-3	FE	W	U	3	89.288
FE-W-S-3	FE	W	S	3	65.281
FE-E-U-3	FE	E	U	3	95.765
FE-E-S-3	FE	E	S	3	42.948
FE-WE-U-3	FE	WE	U	3	84.149
FE-WE-S-3	FE	WE	S	3	40.016
K-W-U-5	K	W	U	5	60.585
K-W-S-5	K	W	S	5	48.777
K-E-U-5	K	E	U	5	63.783
K-E-S-5	K	E	S	5	26.027
K-WE-U-5	K	WE	U	5	56.4761
K-WE-S-5	K	WE	S	5	21.382
F-W-U-5	F	W	U	5	78.763
F-W-S-5	F	W	S	5	55.400
F-E-U-5	F	E	U	5	80.952
F-E-S-5	F	E	S	5	31.007
F-WE-U-5	F	WE	U	5	72.763
F-WE-S-5	F	WE	S	5	30.571
FE-W-U-5	FE	W	U	5	86.446
FE-W-S-5	FE	W	S	5	59.518
FE-E-U-5	FE	E	U	5	91.229
FE-E-S-5	FE	E	S	5	40.779
FE-WE-U-5	FE	WE	U	5	80.891
FE-WE-S-5	FE	WE	S	5	39.580

Table S5b. DPPH — pooled summaries by factor (mean ± SD, n = 18 per level, pooling across the other three factors).

Treatment

Treatment	Mean	SD	n
F	64.069	22.589	18
FE	69.447	22.356	18
K	57.678	22.4328	18

Day

Day	Mean	SD	n
0	70.454	22.387	18
3	63.801	22.495	18
5	56.941	21.975	18

Solvent

Solvent	Mean	SD	n
E	62.830	27.240	18
W	71.836	13.771	18
WE	56.529	23.017	18

Method

Method	Mean	SD	n
S	44.882	13.564	27
U	82.581	10.750	27

Table S5c. DPPH — four-way factorial ANOVA (Treatment × Solvent × Method × Day, with two-way interactions).

Source	SS	df	F	p
C(Treatment)	1249.66	2	121.2	<0.001
C(Solvent)	2130.59	2	206.64	<0.001
C(Method)	19187.12	1	3721.86	<0.001
C(Day)	1643.55	2	159.41	<0.001
C(Treatment):C(Solvent)	4.26	4	0.21	0.933
C(Treatment):C(Method)	86.97	2	8.44	0.001
C(Treatment):C(Day)	474.43	4	23.01	<0.001
C(Solvent):C(Method)	2013.53	2	195.29	<0.001
C(Solvent):C(Day)	5.99	4	0.29	0.882
C(Method):C(Day)	35.77	2	3.47	0.045

K, uncoated fruit (control); F, coated fruit with SA:G; FE, coated fruit with SA:G:LEO. W, water; E, ethanol; WE, water–ethanol (50:50, v/v). U, ultraturrax; S, sonication.

Table S6a. Total phenolic content (mg GAE/100 g fw) — full 54-sample factorial matrix.

Sample code	Treatment	Solvent	Method	Day	TPC (mg GAE/100 g fw)
K-W-U-0	K	W	U	0	449.659
K-W-S-0	K	W	S	0	271.763
K-E-U-0	K	E	U	0	484.557
K-E-S-0	K	E	S	0	146.858
K-WE-U-0	K	WE	U	0	402.725
K-WE-S-0	K	WE	S	0	168.811
F-W-U-0	F	W	U	0	449.697
F-W-S-0	F	W	S	0	272.717
F-E-U-0	F	E	U	0	484.351
F-E-S-0	F	E	S	0	146.494
F-WE-U-0	F	WE	U	0	402.48
F-WE-S-0	F	WE	S	0	168.671
FE-W-U-0	FE	W	U	0	451.903
FE-W-S-0	FE	W	S	0	271.726
FE-E-U-0	FE	E	U	0	485.455
FE-E-S-0	FE	E	S	0	147.391
FE-WE-U-0	FE	WE	U	0	407.607
FE-WE-S-0	FE	WE	S	0	168.466
K-W-U-3	K	W	U	3	428.190
K-W-S-3	K	W	S	3	249.651
K-E-U-3	K	E	U	3	450.219
K-E-S-3	K	E	S	3	137.297
K-WE-U-3	K	WE	U	3	396.818
K-WE-S-3	K	WE	S	3	126.979
F-W-U-3	F	W	U	3	438.304
F-W-S-3	F	W	S	3	263.035
F-E-U-3	F	E	U	3	482.210
F-E-S-3	F	E	S	3	135.373
F-WE-U-3	F	WE	U	3	398.229
F-WE-S-3	F	WE	S	3	165.064
FE-W-U-3	FE	W	U	3	439.126
FE-W-S-3	FE	W	S	3	264.138
FE-E-U-3	FE	E	U	3	483.099
FE-E-S-3	FE	E	S	3	137.317
FE-WE-U-3	FE	WE	U	3	401.744
FE-WE-S-3	FE	WE	S	3	169.354
K-W-U-5	K	W	U	5	402.994
K-W-S-5	K	W	S	5	211.960
K-E-U-5	K	E	U	5	431.798
K-E-S-5	K	E	S	5	111.877
K-WE-U-5	K	WE	U	5	375.386
K-WE-S-5	K	WE	S	5	106.540
F-W-U-5	F	W	U	5	421.358
F-W-S-5	F	W	S	5	258.829
F-E-U-5	F	E	U	5	462.528
F-E-S-5	F	E	S	5	132.485
F-WE-U-5	F	WE	U	5	385.753
F-WE-S-5	F	WE	S	5	151.438
FE-W-U-5	FE	W	U	5	432.929
FE-W-S-5	FE	W	S	5	258.072
FE-E-U-5	FE	E	U	5	478.266
FE-E-S-5	FE	E	S	5	133.242
FE-WE-U-5	FE	WE	U	5	398.211
FE-WE-S-5	FE	WE	S	5	160.447

Table S6b. TPC — pooled summaries by factor (mean ± SD, n = 18 per level, pooling across the other three factors).

Treatment

Treatment	Mean	SD	n
F	312.168	136.072	18
FE	316.0278	137.627	18
K	297.449	139.304	18

Day

Day	Mean	SD	n
0	321.186	137.045	18
3	309.231	137.731	18
5	295.229	137.687	18

Solvent

Solvent	Mean	SD	n
E	303.934	172.971	18
W	346.4478	92.578	18
WE	275.262	125.936	18

Method

Method	Mean	SD	n
S	182.815	57.347	27
U	434.281	34.496	27

Table S6c. TPC — four-way factorial ANOVA (Treatment × Solvent × Method × Day, with two-way interactions).

Source	SS	df	F	p
C(Treatment)	3460.13	2	28.99	<0.001
C(Solvent)	46180.43	2	386.85	<0.001
C(Method)	853679.49	1	14302.55	<0.001
C(Day)	6076.28	2	50.9	<0.001
C(Treatment):C(Solvent)	41.35	4	0.17	0.950
C(Treatment):C(Method)	101.41	2	0.85	0.438
C(Treatment):C(Day)	2133.4	4	8.94	<0.001
C(Solvent):C(Method)	56693.28	2	474.92	<0.001
C(Solvent):C(Day)	85.25	4	0.36	0.837
C(Method):C(Day)	5.7	2	0.05	0.953

K, uncoated fruit (control); F, coated fruit with SA:G; FE, coated fruit with SA:G:LEO. W, water; E, ethanol; WE, water–ethanol (50:50, v/v). U, ultraturrax; S, sonication.

Table S7a. Individual phenolic compounds (mg/100 g fw) — full 54 × 14 factorial matrix.

Sample	Catechin	Epicatechin	EGC	PB1	PB2	Gallic	Ferulic	Chlorogenic	Caffeic	NOCH.	p-Coumaric	Q-3-G	K-3-G	Rutin	Trt	Solv	Meth	Day
K-W-U-0	9.111	0.7412	3.69511	2.3325	2.08448	3.4367	0.9371	1.4641	0.3545	0.4509	1.7786	1.1283	0.5425	0.52999	K	W	U	0
K-W-S-0	6.948	0.6659	3.14113	2.0936	1.15442	2.4359	0.7957	1.1801	0.2213	0.3581	1.6802	1.0994	0.4467	0.45325	K	W	S	0
K-E-U-0	10.493	1.0027	4.33445	2.5898	2.13573	3.5564	1.1204	1.576	0.4773	0.5393	1.8101	1.1745	0.8944	0.71058	K	E	U	0
K-E-S-0	4.493	0.973	2.28321	1.4872	0.73161	2.2235	0.6761	1.1286	0.0167	0.2166	1.4669	1.0091	0.3402	0.31345	K	E	S	0
K-WE-U-0	8.765	0.8756	3.4657	2.3094	1.9687	3.4148	0.9125	1.4543	0.3231	0.4213	1.7275	1.1023	0.4659	0.5042	K	WE	U	0
K-WE-S-0	5.453	0.4534	2.1975	1.0445	0.6284	2.3753	0.4856	1.1053	0.1156	0.2031	1.4545	1.0024	0.2261	0.3059	K	WE	S	0
F-W-U-0	9.353	0.9407	3.6929	2.3572	2.0684	3.4371	0.9378	1.4616	0.3564	0.4573	1.7767	1.1295	0.5437	0.5367	F	W	U	0
F-W-S-0	6.569	0.7678	3.1426	2.0996	1.1515	2.4372	0.793	1.1814	0.2234	0.3593	1.6845	1.0197	0.4487	0.4544	F	W	S	0
F-E-U-0	11.001	0.9924	4.3317	2.5873	2.1347	3.5546	1.1277	1.5761	0.4795	0.5355	1.8123	1.1749	0.8968	0.7113	F	E	U	0
F-E-S-0	4.01074	0.5716	2.2883	1.4822	0.7391	2.2246	0.6723	1.1235	0.0169	0.2127	1.4674	1.0096	0.3434	0.3241	F	E	S	0
F-WE-U-0	8.9945	0.8761	3.4645	2.3084	1.9675	3.4176	0.9126	1.4578	0.2245	0.4213	1.7287	1.1035	0.5062	0.5055	F	WE	U	0
F-WE-S-0	4.0014	0.4541	2.1928	1.0439	0.6753	2.3733	0.4854	1.1104	0.1195	0.2078	1.4547	1.0024	0.2265	0.3062	F	WE	S	0
FE-W-U-0	9.7572	0.9492	3.6998	2.3381	2.0886	3.4389	0.9351	1.4619	0.3597	0.4584	1.7769	1.1296	0.545	0.5372	FE	W	U	0
FE-W-S-0	6.73979	0.7696	3.1466	2.0892	1.1502	2.4356	0.7928	1.1812	0.2245	0.3598	1.6847	1.0197	0.4489	0.4546	FE	W	S	0
FE-E-U-0	10.7441	0.9969	4.3309	2.5815	2.1348	3.5548	1.1251	1.5739	0.4799	0.5399	1.8125	1.1746	0.8972	0.7125	FE	E	U	0
FE-E-S-0	4.84417	0.5721	2.2849	1.4824	0.7312	2.2265	0.6712	1.1282	0.0162	0.2167	1.4679	1.1004	0.3442	0.3271	FE	E	S	0
FE-WE-U-0	8.9856	0.8798	3.4654	2.3092	1.9653	3.4123	0.9124	1.4578	0.2253	0.4219	1.7283	1.1037	0.5071	0.5067	FE	WE	U	0
FE-WE-S-0	4.7689	0.4557	2.1963	1.0419	0.6782	2.3756	0.4853	1.1156	0.1197	0.2037	1.4548	1.0025	0.2269	0.3078	FE	WE	S	0
K-W-U-3	3.2295	0.16516	1.1095	1.0678	1.0196	2.34253	0.3182	0.7945	0.0135	0.0986	1.0146	1.0045	0.1265	0.1268	K	W	U	3
K-W-S-3	1.4017	0.0669	1.0015	0.7111	0.7179	1.3918	0.1183	0.6756	0.0056	0.0547	0.9446	0.9456	0.0954	0.0059	K	W	S	3
K-E-U-3	5.7287	0.32733	1.2265	1.1874	1.0992	2.1234	0.5034	0.8889	0.0546	0.1431	1.0456	1.0924	0.1483	0.1481	K	E	U	3
K-E-S-3	2.0010	0.0556	1.00544	0.3705	0.5435	1.1755	0.0935	0.6054	0.0021	0.0034	0.7535	0.8657	0.0759	0.0052	K	E	S	3
K-WE-U-3	2.6578	0.0737	1.0448	1.0362	1.0042	2.3425	0.2648	0.7094	0.0085	0.0978	1.3237	1.0033	0.1067	0.1092	K	WE	U	3
K-WE-S-3	1.0453	0.0123	0.9749	0.0767	0.8539	1.0126	0.0737	0.5775	0.0015	0.0027	0.6536	0.9347	0.0526	0.0034	K	WE	S	3
F-W-U-3	9.2056	0.7165	3.5643	2.2235	1.9753	3.2467	0.9045	1.3754	0.3145	0.4025	1.5647	1.0945	0.4979	0.4962	F	W	U	3
F-W-S-3	6.4272	0.6692	2.33461	1.7754	1.1207	1.93109	0.529	1.1254	0.1934	0.2298	1.2535	1.0154	0.3254	0.3867	F	W	S	3
F-E-U-3	10.8056	0.7545	4.3095	2.4829	2.1035	3.4852	1.0665	1.5567	0.4256	0.5063	1.7835	1.1056	0.8653	0.6826	F	E	U	3
F-E-S-3	3.9135	0.5747	1.40893	1.3592	0.5302	1.8065	0.4359	1.0717	0.0146	0.1564	1.1256	0.9741	0.2961	0.2769	F	E	S	3
F-WE-U-3	8.7123	0.6849	2.2159	2.2563	1.8764	3.1245	0.8725	1.3245	0.2065	0.3765	1.4567	1.1031	0.4853	0.4845	F	WE	U	3
F-WE-S-3	3.8212	0.4131	1.0437	1.0036	0.1864	1.5764	0.2765	1.0464	0.0956	0.1497	1.1046	0.9567	0.2069	0.2136	F	WE	S	3
FE-W-U-3	9.5642	0.7865	3.6594	2.3176	1.9636	3.3256	0.9154	1.4146	0.3125	0.4178	1.6235	1.1045	0.5147	0.5067	FE	W	U	3
FE-W-S-3	6.6204	0.6696	3.0541	1.9793	1.1396	2.0456	0.562	1.1524	0.0196	0.2863	1.4867	1.0162	0.4275	0.4254	FE	W	S	3
FE-E-U-3	10.6522	0.8946	4.3141	2.5209	2.1017	3.5173	1.0765	1.4835	0.4534	0.5168	1.7934	1.1535	0.8851	0.6947	FE	E	U	3
FE-E-S-3	4.82826	0.5992	2.1651	1.4134	0.6213	1.9843	0.4843	1.1353	0.0159	0.1664	1.1437	0.9952	0.2958	0.3031	FE	E	S	3
FE-WE-U-3	8.7538	0.7524	3.4227	2.2928	1.9037	3.2543	0.9035	1.3845	0.2087	0.4104	1.6056	1.1024	0.4462	0.4851	FE	WE	U	3
FE-WE-S-3	4.5124	0.4264	2.0931	1.0152	0.2321	1.9756	0.3854	1.1076	0.1069	0.1591	1.4046	1.002	0.2082	0.2712	FE	WE	S	3
K-W-U-5	1.01153	0.02838	0.3407	0.0611	0.2014	0.4505	0.0621	0.1643	0.0044	0.0021	0.0047	0.0846	0.0024	0.0057	K	W	U	5
K-W-S-5	0.42642	0.0019	0.0028	0.0046	0.0087	0.2132	0.0073	0.0045	0.0012	0.0011	0.0016	0.0437	0.0008	0.0026	K	W	S	5
K-E-U-5	2.25156	0.05791	0.5416	0.2348	0.337	0.5003	0.1166	0.4764	0.0034	0.0065	1.0045	0.1267	0.0065	0.0081	K	E	U	5
K-E-S-5	0.52929	0.0022	0.0058	0.0044	0.0054	0.1235	0.0042	0.0037	0.0006	0.0005	0.0006	0.0246	0.0004	0.0016	K	E	S	5
K-WE-U-5	0.2658	0.0046	0.1438	0.0345	0.0138	0.4326	0.0234	0.0084	0.0037	0.0053	0.0026	0.0672	0.0014	0.0045	K	WE	U	5
K-WE-S-5	2.0216	0.0005	0.0018	0.0015	0.0021	0.1026	0.0026	0.0032	0.0005	0.0002	0.0005	0.0068	0.0002	0.0014	K	WE	S	5
F-W-U-5	9.09217	0.6782	3.4598	2.1515	1.8946	3.2069	0.8582	1.3223	0.2994	0.3734	1.5057	0.9769	0.4582	0.4861	F	W	U	5
F-W-S-5	6.4014	0.54213	1.9733	1.4371	0.9455	1.7873	0.4704	1.11754	0.1792	0.1976	1.2169	0.8642	0.2354	0.3245	F	W	S	5
F-E-U-5	10.6177	0.6804	4.2707	2.3802	2.0958	3.4325	1.0077	1.4234	0.3742	0.4596	1.7536	1.0004	0.8263	0.6267	F	E	U	5
F-E-S-5	3.8689	0.36674	1.1328	1.1417	0.5122	1.7528	0.3887	1.0336	0.0138	0.1145	1.1047	0.8472	0.2032	0.2554	F	E	S	5
F-WE-U-5	8.6245	0.6087	1.9537	2.5256	1.8237	3.1065	0.7954	1.2653	0.1854	0.3525	1.4127	0.9661	0.4257	0.4546	F	WE	U	5
F-WE-S-5	3.7819	0.3599	1.0038	0.7247	0.1253	1.5529	0.2532	1.0046	0.0946	0.1246	1.0947	0.8223	0.1845	0.2061	F	WE	S	5
FE-W-U-5	9.53872	0.7585	3.4732	2.2583	1.9101	3.2776	0.8792	1.3734	0.2974	0.3979	1.6068	1.0037	0.4756	0.4837	FE	W	U	5
FE-W-S-5	6.70482	0.6419	2.87786	1.6535	1.0299	1.9998	0.4993	1.1146	0.018	0.2197	1.3725	0.9671	0.3661	0.3864	FE	W	S	5
FE-E-U-5	10.6319	0.8193	4.2983	2.4654	2.0989	3.4747	1.0276	1.4545	0.3798	0.4875	1.7567	1.1234	0.8657	0.6571	FE	E	U	5
FE-E-S-5	4.75453	0.5804	1.9784	1.1547	0.5939	1.8458	0.4089	1.1036	0.0136	0.1498	1.1263	0.8872	0.2746	0.2769	FE	E	S	5
FE-WE-U-5	8.6751	0.7365	3.3884	2.2385	1.8548	3.2131	0.8235	1.3343	0.1967	0.3735	1.5705	1.0012	0.4048	0.4638	FE	WE	U	5
FE-WE-S-5	4.3848	0.4139	1.8599	0.9327	0.2063	1.8361	0.3143	1.0846	0.1065	0.1476	1.2451	0.8346	0.1835	0.2437	FE	WE	S	5

Table S7b. Per-compound ANOVA (main effects of Treatment, Solvent, Method, Day; n = 54 per compound).

Compound	Treatment F	Treatment p	Solvent F	Solvent p	Method F	Method p	Day F	Day p
Catechin	24.17	<0.001	2.08	0.137	59.96	<0.001	8.06	<0.001
Epicatechin	29.47	<0.001	3.21	0.050	21.16	<0.001	24.13	<0.001
Epigallocatechin	23.26	<0.001	3.99	0.025	35.77	<0.001	16.38	<0.001
Procyanidin B1	26.47	<0.001	3.27	0.047	47.29	<0.001	13.85	<0.001
Procyanidin B2	11.91	<0.001	3.09	0.055	105.86	<0.001	10.6	<0.001
Gallic acid	24.33	<0.001	0.23	0.796	70.38	<0.001	20.88	<0.001
Ferulic Acid	27.67	<0.001	4.27	0.020	68.02	<0.001	24.66	<0.001
Chlorogenic Acid	32.09	<0.001	0.78	0.466	20.59	<0.001	17.14	<0.001
Caffeic Acid	8	0.001	1.78	0.181	46.97	<0.001	7.74	0.001
Neochlorogenic acid	23.98	<0.001	2.26	0.116	63.02	<0.001	19.34	<0.001
p-Coumaric Acid	21.84	<0.001	0.58	0.563	16.17	<0.001	21.94	<0.001
Quercetin-3-glicosid	14.28	<0.001	0.18	0.835	4.06	0.050	23.55	<0.001
Keampherol-3-glicosid	17.13	<0.001	7.79	0.001	37.69	<0.001	9.85	<0.001
Rutin	28.69	<0.001	2.94	0.063	37.77	<0.001	14.47	<0.001

K, uncoated fruit (control); F, coated fruit with SA:G; FE, coated fruit with SA:G:LEO. W, water; E, ethanol; WE, water–ethanol (50:50, v/v). U, ultraturrax; S, sonication. EGC, epigallocatechin; PB1/PB2, procyanidin B1/B2; Q-3-gluc, quercetin-3-glucoside; K-3-gluc, kaempferol-3-glucoside.

Table S8. Hyperparameters and apparent fit (R^2, full dataset) of the final SVR–RBF model for each phenolic compound (n = 54).

**Target**	**Hyperparameters**			**R^2**
	**C**	ε	γ	**(full)**
Ferulic Acid	10	0.01	0.1	0.998
Chlorogenic Acid	1000	0.01	0.01	0.992
Catechin	100	0.01	0.1	0.988
Neochlorogenic Acid	10	0.01	auto	0.997
Gallic Acid	100	0.1	0.1	0.992
Procyanidin B1	10	0.01	0.1	0.989
Rutin	10	0.01	auto	0.998
Procyanidin B2	100	0.01	auto	0.999
Epigallocatechin	10	0.01	scale	1.000
Epicatechin	1000	0.01	0.01	0.966
Kaempferol-3-glucoside	10	0.01	scale	0.999
Caffeic Acid	1	0.01	scale	0.997
p-Coumaric Acid	100	0.01	0.01	0.918
Quercetin-3-glucoside	100	0.01	0.1	0.999

These hyperparameters correspond to the final model fit on the full dataset, used only for the permutation-based feature importance analysis (Fig. 8). They are distinct from the fold-specific hyperparameters selected independently within each outer fold of the nested cross-validation, which are not reported as a single fixed set (see Section 2.12) and underlie the performance metrics in Table 1. R^2 (full dataset) is the apparent fit of the final model on all 54 samples and is shown only to illustrate the optimism of non-nested estimates; it must not be interpreted as predictive performance.
